# PACLseq: A Standalone Diagnostic Method for Ph‐Like Acute Lymphoblastic Leukemia Using Nanopore Sequencing

**DOI:** 10.1002/mco2.70360

**Published:** 2025-09-16

**Authors:** Hang Zhang, Huan Yu, Yanmei Chen, Kai Jiang, Beibei Huo, Jialin Li, Ting Liu, Dan Xie

**Affiliations:** ^1^ Department of Hematology, Institute of Hematology West China Hospital of Sichuan University Chengdu Sichuan China; ^2^ Qitan Technology Ltd. Chengdu Sichuan China; ^3^ Laboratory of Omics Technology and Bioinformatics, Frontiers Science Center for Disease‐related Molecular Network, State Key Laboratory of Biotherapy West China Hospital Sichuan University Chengdu Sichuan China

**Keywords:** gene fusion, long‐read sequencing, Philadelphia chromosome–like acute lymphoblastic leukemia, Target transcriptome sequencing

## Abstract

Timely and accurate detection of Philadelphia chromosome–like acute lymphoblastic leukemia (Ph‐like ALL)‐related fusion gene is essential for treatment decisions. However, due to the complexity of possible gene fusion combinations of Ph‐like ALL, current diagnostic workflows face critical limitations: prolonged turnaround (7–14 days), high costs, and deficiency in degraded specimens. In this study, we introduce Partial Anchored Capture and Long‐Read Sequencing (PACLseq), a nanopore‐sequencing‐technology‐based approach. We designed a detection panel associated with Ph‐like ALL, specifically *ABL2*, *CSF1R*, *PDGFRB*, *JAK2*, *ABL1*, *EPOR*, and *CRLF2* as target genes. Validated on 47 clinical samples, PACLseq achieved 93.3% sensitivity and 100% specificity in 26 degraded RNA samples (RIN > 3). Crucially, PACLseq maintained detection accuracy in nine low‐RIN samples (RIN ≤ 3) with fragmented transcripts. The method requires only 10 ng of RNA input, delivers results in 3 days (vs. 7–14 days for conventional methods), and reduces costs by 50%. By offering rapid and accurate fusion detection, PACLseq has the potential to significantly improve diagnostic efficiency, facilitate timely treatment decisions, and enhance patient outcomes in the management of Ph‐like ALL.

## Graphical abstract

PACLseq has demonstrated reliable detection results with a sensitivity of 93.33% and specificity of 100%, along with a short turnaround time, cost‐effectiveness, a streamlined workflow, and resilience towards poor RNA quality and small sample input. Consequently, it proves to be an excellent choice for diagnosis of Ph‐like ALL in clinical settings.

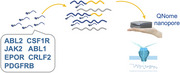

## 1 Introduction

Philadelphia chromosome‐like acute lymphoblastic leukemia (Ph‐like ALL) was initially described in 2009 by Mullighan's group in the United States [1] and Den Boer's group in the Netherlands [2]. These two research groups independently identified a novel subtype of ALL that lacked the Philadelphia chromosome (*BCR::ABL1* fusion) but exhibited gene expression profiles similar to Philadelphia chromosome‐positive ALL (Ph+ ALL). Ph‐like ALL is seen in 10%–20% of pediatric cases and 20%–30% of adult cases of ALL and is associated with a high rate of relapse and poor prognosis. In 2022, both the World Health Organization (WHO) and the International Consensus Classification officially classified Ph‐like ALL as a distinct high‐risk subtype of B‐cell acute lymphoblastic leukemia (B‐ALL) [3, 4].

Ph‐like ALL is characterized by highly complex genomic alterations, primarily involving fusion genes. These fusions can be classified into the following subsets: ABL family (ABL1, ABL2, CSF1R, and PDGFRB), JAK/STAT pathway‐related genes (CRLF2, JAK2, and EPOR), and other kinases (BLNK, DGKH, FGFR1, IL2RB, LYN, NTRK3, PDGFRA, PTK2B, and TYK2) [5]. Each of these genes may have different breakpoints and partner genes, leading to distinct fusion transcripts. So far, a total of 95 fusions have been reported (Table S1), and it is anticipated that more fusions will be discovered in the future with the advancement of next‐generation sequencing (NGS) technologies. The detection of fusions serves not only as a diagnostic tool but also as a foundation for subsequent targeted therapy decisions. In Ph‐like ALL, the patients with ABL‐derived may benefit from tyrosine kinase inhibitors (such as dasatinib), and those with JAK–STAT‐derived, from the addition of JAK inhibitors (such as ruxolitinib) to chemotherapy regimens [6, 7, 8, 9, 10, 11, 12, 13, 14].

Accurate diagnosis and timely initiation of targeted therapy are critical for the effective management of Ph‐like ALL at initial presentation. Currently, three diagnostic protocols are employed for Ph‐like ALL [15]: (1) The combination of flow cytometry (detection for *CRLF2* overexpression) and fluorescence in situ hybridization (FISH) method allows for the cost‐effective detection of a specific diagnostic panel. (2) Calculation of gene expression coefficients using a quantitative real‐time reverse transcription polymerase chain reaction (RT‐PCR)‐based low‐density array (LDA) platform, followed by downstream pathway analysis in screened‐positive patients [16]. (3) The application of NGS techniques, such as whole exome sequencing, whole genome sequencing, or whole transcriptome sequencing (RNA‐seq) [17], enables the comprehensive acquisition of molecular genomic information. However, these methods are associated with certain limitations: incomplete detection of fusion genes, procedural complexity, high cost, and long diagnostic turnaround times extending over several weeks. These limitations are inadequate for the imperative need of early diagnosis and timely targeted treatment of Ph‐like ALL. Therefore, the development of a rapid and accurate diagnostic approach applicable within individual clinical setting is of paramount importance for Ph‐like ALL management.

Targeted RNA sequencing represents a promising and cost‐effective strategy for clinical applications, which targets either one partner or a partial short sequence of a transcript and has the potential to sequence the full‐length transcript, thereby enhancing the detection of new fusion transcripts. Moreover, long‐read sequencing may contribute a better alignment and fusion detection performance [18, 19]. The nanopore long‐read sequencing platform also offers advantages such as small size, simple library preparation, minimal capital cost, and reduced turnaround time. Therefore, to streamline the diagnostic approach for Ph‐like ALL and capitalize on the advantages of long‐read sequencing to identify fusions, including novel fusion partners, we introduce an innovative methodology termed partial anchored capture and long‐read sequencing (PACLseq) based on QitanTech QNome nanopore sequence platform. This method facilitates the targeted analysis of a specific gene panel while simultaneously providing the opportunity to identify all fusion partner genes of Ph‐like ALL in a timely fashion. We comprehensively evaluated the diagnostic prowess of PACLseq for the detection of fusions associated with Ph‐like ALL.

## 2 Results

### 2.1 PACLseq Method Establishment and Test

**FIGURE 1:**
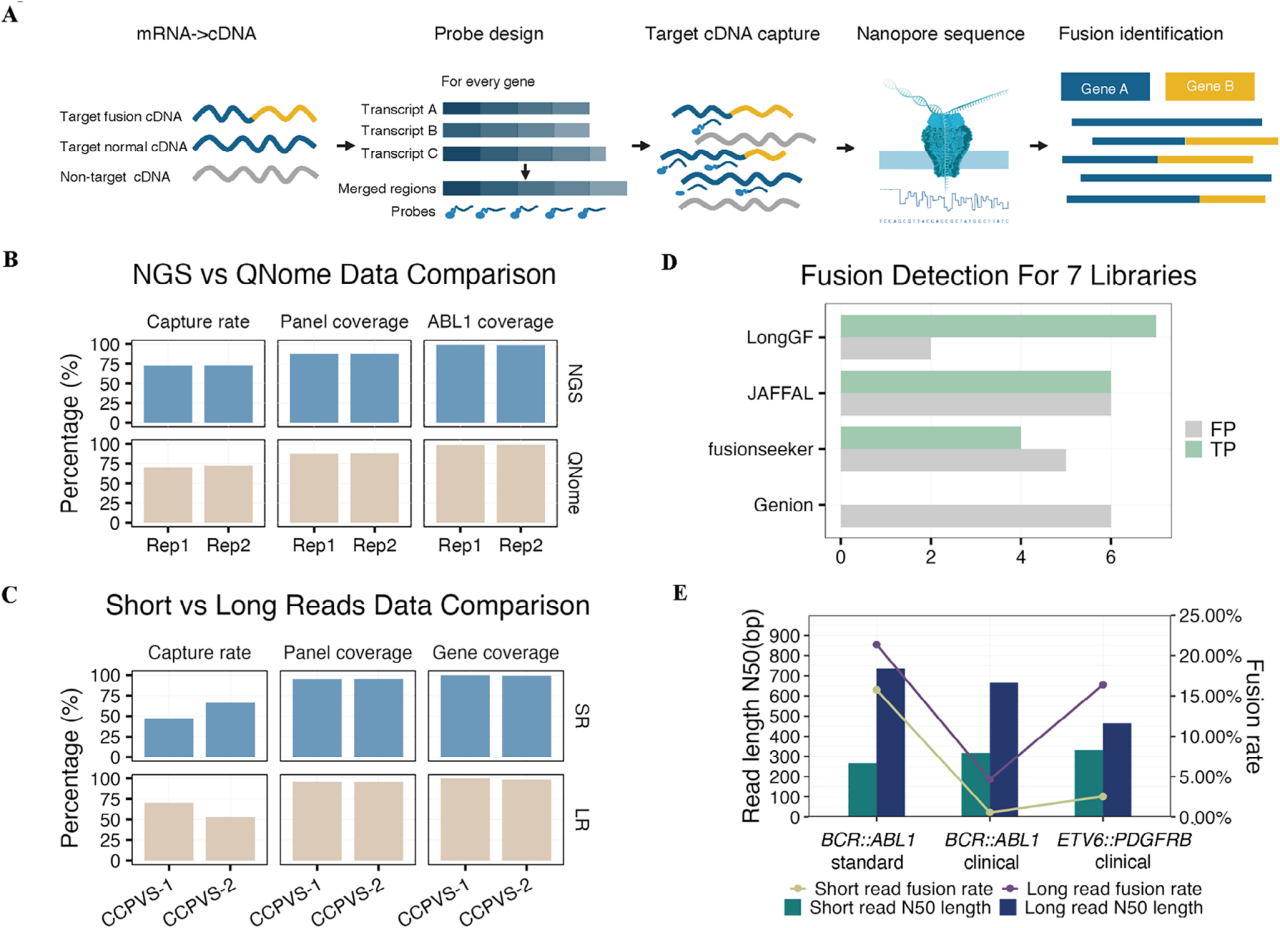
PACLseq scheme and performance. (A) Schematic overview of the PACLseq methodology. Biotinylated probes targeting pan‐transcriptomic regions enable cDNA capture and enrichment of fusion transcripts prior to nanopore sequencing. (B) Cross‐platform validation using *BCR::ABL1* positive standards (rep1 and rep2 for replicates). Comparative analysis of capture efficiency, target region coverage, and ABL1‐specific coverage between Illumina/NGS (top) and QNome nanopore platforms (bottom). (C) Library fragment size optimization. Performance metrics (capture rate, target coverage, and fusion‐specific coverage) for *BCR::ABL1* (CCPVS‐2) and *ETV6::PDGFRB* (CCPVS‐1) clinical samples using short‐fragment (SR, top) versus long‐fragment (LR, bottom) libraries on QNome. (D) Fusion detection benchmarking across seven libraries. True positives (TP) and false positives (FP) were evaluated using four algorithms: GenION, JAFFAL, FusionSeeker, and LongGF. LongGF achieved 100% sensitivity with the lowest false positive rate. (E) Positive correlation between read length and fusion detection frequency observed in: *BCR::ABL1* positive standard (left), *BCR::ABL1 positive* clinical sample (middle), and *ETV6::PDGFRB positive* clinical sample (right).

PACLseq captures a partial region of a long DNA fragment (Figure 1A, Figure S1). If there are gene fusion events associated with any of the genes in the panel, the nanopore sequencing technology would read through the fusion junction and allow us to identify the other partner of the fusion event. Two short‐fragment hybridization capture libraries (270–350 bp) were constructed using the commercial *BCR::ABL1* standard sample and subsequently sequenced using Illumina and QNome nanopore platforms. Both platforms successfully detected *BCR::ABL1* fusion, highlighting the effective capture performance of the panel (Figure 1B, Tables S3 and S4).

### 2.2 Validation of PACLseq and Bioinformatics Method Establishment

We conducted validation experiments using two clinically confirmed fusion‐positive samples, one *BCR::ABL1* fusion (CCPVS‐2) and one *ETV6::PDGFRB* fusion (CCPVS‐1) (Figure 1C, Table 1) as well as the commercial *BCR::ABL1* standard sample. To simultaneously determine the most suitable bioinformatics pipeline, we assessed several candidate software tools, including LongGF [20], JAFFAL [21], Genion [22], and Fusionseeker [23]. After testing these methods on seven libraries (three long‐fragment and four short‐fragment libraries), we found that LongGF exhibited the highest sensitivity of 100%. However, all four methods demonstrated some degree of false‐positive fusions detection (Figure 1D, Table S5). To optimize the analysis process and reduce false‐positive results, we then developed an optimized bioinformatics pipeline based on LongGF, which effectively identified fusion events detected by PACLseq while minimizing false positives (see Supporting Information).

**TABLE 1:** Basic information about clinically confirmed positive validation samples.

Sample ID	Sample type	Sex	Diagnosis	Disease status	Clinical fusion result	Clinical fusion frequency
CCPVS‐1	PB	Male	CMML	First diagnosed	*ETV6::PDGFRB*	Not available
CCPVS‐2	BM	Female	B‐ALL	PR	*BCR::ABL1*	32.41%
CCPVS‐3	PB	Female	CML	Chronic phase	*BCR::ABL1*	0.06%
CCPVS‐4	BM	Female	CML	Chronic phase	*BCR::ABL1*	2.91%
CCPVS‐5	PB	Male	CML	Chronic phase	*BCR::ABL1*	9.82%
CCPVS‐6	PB	Male	CML	Chronic phase	*BCR::ABL1*	0.09%
CCPVS‐7	BM	Male	CML	Chronic phase	*BCR::ABL1*	3.49%
CCPVS‐8	PB	Female	CML	Chronic phase	*BCR::ABL1*	32.65%

Abbreviations: B‐ALL: B‐cell acute lymphoblastic leukemia; BM, bone marrow; CMML, chronic myelomonocytic leukemia; CML, chronic myeloid leukemia; PB, peripheral blood; PR, partial remission.

Applying the optimized bioinformatics pipeline, we achieved favorable coverage performance for both short‐fragment and long‐fragment libraries of the three samples mentioned above. Capture rates ranged from 47.23% to 70.38%, with coverage of the target region ranging from 90.83% to 96.1% (Figure 1C, Table S3). Moreover, we observed that long‐fragment sequencing outperformed short‐fragment sequencing in fusion detection, showing a 5.28‐fold higher detection rate (Figure 1E, Table S4).

### 2.3 Evaluation of Minimal cDNA Input

**FIGURE 2:**
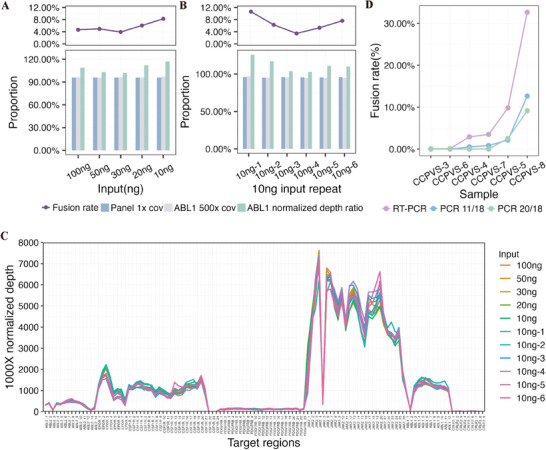
Optimization of experimental parameters. (A) Determination of minimal cDNA input. A BCR::ABL1‐positive clinical sample (CCPVS‐2) was tested with cDNA inputs of 100, 50, 30, 20, and 10 ng. Results showed comparable data quality (1x coverage, target gene ABL1 500x coverage, ABL1 normalized depth ratio compared to average depth) at 10 ng. (B) Reproducibility assessment: Six replicates with 10 ng input demonstrated consistent quality control performance. (C) Coverage depth distribution: Normalized coverage depth at 1000x average depth showed similar trends across all input libraries. (D) PCR cycle optimization: Using RT‐PCR as the reference standard, fusion detection performance across six additional clinical samples supported the use of 11–18 PCR cycles as the optimal range.

To identify the optimal cDNA input, a clinically confirmed positive validation sample (CCPVS‐2) was utilized, and different cDNA input levels were compared, including 100 ng as the control and 50, 30, 20, and 10 ng as the test groups. All tested cDNA input levels successfully detected *BCR::ABL1* fusion without any false positives (Figure 2A, Tables S3 and S4).

To further evaluate the performance of the 10 ng cDNA input, additional six libraries with 10 ng input cDNA were constructed using different polymerase chain reaction (PCR) cycles. These libraries consistently yielded *BCR::ABL1* fusion results (Figure 2B, Tables S3 and Table S4), and the normalized depth at 1000x (1000 × target gene average depth/panel average depth) for all 11 libraries exhibited a remarkably uniform distribution (Figure 2C). Among the tested combinations, the one with 11 pre‐capture cycles and 18 post‐capture cycles (simplified as 11/18) achieved the highest fusion frequency at 10.63%, while other combinations yielded approximately 5% fusion frequency.

### 2.4 Evaluation of Optimal PCR Conditions and Detection Limit of Fusion Frequency

We proceeded to evaluate the optimal pre‐capture and post‐capture PCR cycles, as well as the fusion frequency detection limit, using six clinically confirmed positive validation samples with fusion frequencies of 0.06%, 0.09%, 2.91%, 3.49%, 9.82%, and 32.65% (basic information shown in Table 1). Based on the outcomes obtained from the preceding step, we categorized the samples into two groups. One group underwent PCR cycles 11/18, while the other group underwent PCR cycles 20/18.

The results demonstrated that the PCR cycle combination 11/18 detected four (4/6) fusions, with the lowest RT‐PCR detected fusion frequency being 2.91% (0.72 million reads) in the clinical setting. In contrast, the PCR cycle combination 20/18 only detected two out of six fusions (Figure 2D, Table S4). This further confirms the suitability of 10 ng input cDNA in the PACLseq method, and we determined the PCR cycle combination to be 11/18. Based on our results, we hypothesize that our method can qualitatively detect fusion at a fusion frequency as low as a 10^−2^ level.

### 2.5 Blind Test Validation Using Clinical Samples

After configuring the key parameters in our method, including a fragment size of ≥ 200 bp, a cDNA input of 10 ng, and a PCR cycle combination of 11/18, we conducted a blind test using clinical blind‐test samples. Given that the current methodology of PACLseq can only identify mRNA sequences, our primary focus was on fusion genes other than those involving *IGH* and *IGK*. This choice was motivated by the fact that fusion genes composed of *IGH* and *IGK* often introduce non‐coding regulatory elements, as well as non‐templated nucleotides at breakpoints [24]. To avoid the influence of the non‐templated region on the detection accuracy of PACLseq, we categorized the clinically confirmed fusion genes whose breakpoints occurred in the coding region into group A and group B based on the RIN values. Specimens with RIN > 3 were assigned to group A, while those with RIN ≤ 3 were designated as group B. While samples containing *IGH*, *IGK*‐related fusions or those with unknown partner genes were separately classified into group C.

**FIGURE 3:**
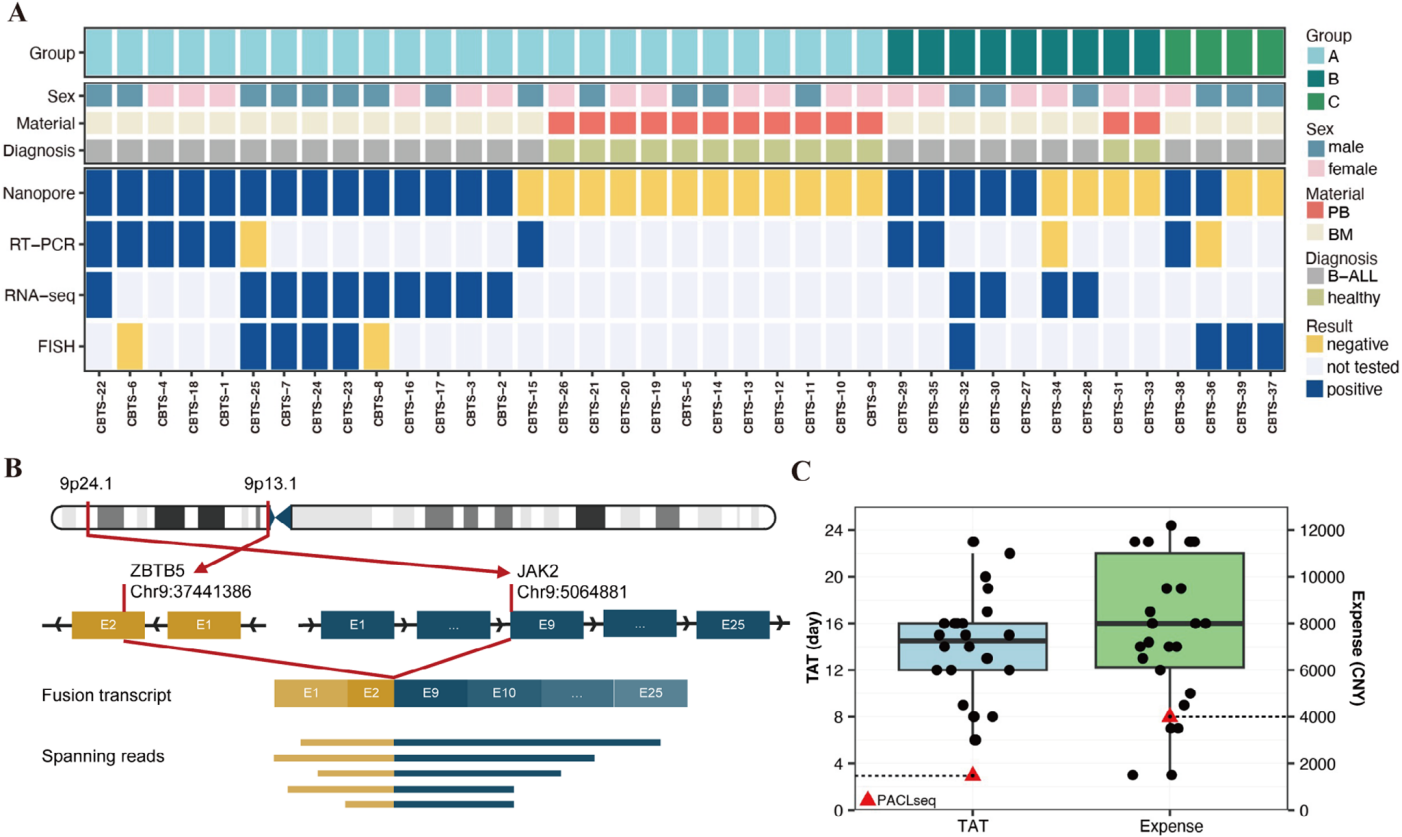
Fusion identification in clinical blind‐test samples. (A) Landscape of fusion gene identification in clinical blind‐test samples, PB: peripheral blood, BM: bone marrow. (B) Structure of novel fusion gene *ZBTB5::JAK2* identified in sample CBTS‐25. (C) The turnaround time and expense of detection of fusion genes. Boxplot: actual turnaround time and expense spent in the clinical setting for each patient. Red triangle: expected turnaround time and expense by PACLseq. TAT: turnaround time.

The blind test included a total of 39 clinical samples, with 26 in group A, nine in group B, and four in group C (Table 2, Figure 3A, Table S6). These samples were sent to the laboratory for sequencing and analysis without revealing any information. The unblinding process was conducted after the acquisition of sequencing results.

**TABLE 2:** Basic information about clinical blind‐test samples.

Sample ID	Group	Sample type	Sex	Diagnosis	PACLseq	RT‐PCR	RNA‐seq	FISH
CBTS‐1	A	BM	Female	B‐ALL	*P2RY8::CRLF2*	*P2RY8::CRLF2*	/	/
CBTS‐18	A	BM	Female	B‐ALL	*P2RY8::CRLF2*	*P2RY8::CRLF2*	/	/
CBTS‐6	A	BM	Male	B‐ALL	*P2RY8::CRLF2*	*P2RY8::CRLF2*	/	—
CBTS‐4	A	BM	Female	B‐ALL	*SSBP2::CSF1R*	*SSBP2::CSF1R*	/	/
CBTS‐22	A	BM	Male	B‐ALL	*NUP214::ABL1*	*NUP214::ABL1*	*NUP214::ABL1*	/
CBTS‐23	A	BM	Male	B‐ALL	*P2RY8::CRLF2*	/	*P2RY8::CRLF2*	Possible rearrangement of *CRLF2*
CBTS‐24	A	BM	Male	B‐ALL	*P2RY8::CRLF2*	/	*P2RY8::CRLF2*	Possible rearrangement of *CRLF2*
CBTS‐2	A	BM	Female	B‐ALL	*P2RY8::CRLF2*	/	*P2RY8::CRLF2*	/
CBTS‐3	A	BM	Female	B‐ALL	*P2RY8::CRLF2* *MYO18B::ABL1*	/	*P2RY8::CRLF2* *MYO18B::ABL1*	/
CBTS‐8	A	BM	Male	B‐ALL	*P2RY8::CRLF2*	/	*P2RY8::CRLF2*	—
CBTS‐17	A	BM	Male	B‐ALL	*P2RY8::CRLF2*	/	*P2RY8::CRLF2*	/
CBTS‐7	A	BM	Female	B‐ALL	*NUP214::ABL1*	/	*NUP214::ABL1*	Possible rearrangement of *ABL1*
CBTS‐16	A	BM	Female	B‐ALL	*PAX5::JAK2*	/	*PAX5::JAK2*	/
CBTS‐25	A	BM	Male	B‐ALL	*ZBTB5::JAK2*	—	*ZBTB5::JAK2*	Possible rearrangement of *JAK2*
CBTS‐15	A	BM	Male	B‐ALL	—	*ETV6::ABL1*	/	/
CBTS‐9	A	PB	Female	healthy	—	—	/	/
CBTS‐10	A	PB	Female	healthy	—	—	/	/
CBTS‐11	A	PB	Male	healthy	—	—	/	/
CBTS‐12	A	PB	Female	healthy	—	—	/	/
CBTS‐13	A	PB	Female	healthy	—	—	/	/
CBTS‐14	A	PB	Male	healthy	—	—	/	/
CBTS‐5	A	PB	Male	healthy	—	—	/	/
CBTS‐19	A	PB	Female	healthy	—	—	/	/
CBTS‐20	A	PB	Female	healthy	—	—	/	/
CBTS‐21	A	PB	Male	healthy	—	—	/	/
CBTS‐26	A	PB	Female	healthy	—	—	/	/
CBTS‐30	B	BM	Male	B‐ALL	*P2RY8::CRLF2*	/	*P2RY8::CRLF2*	/
CBTS‐27	B	BM	Female	B‐ALL	*P2RY8::CRLF2*	/	/	/
CBTS‐29	B	BM	Female	B‐ALL	*EBF1::PDGFRB*	*EBF1::PDGFRB*	/	/
CBTS‐32	B	BM	Male	B‐ALL	*EBF1::PDGFRB*	/	*EBF1::PDGFRB*	Possible rearrangement of *PDGFRB* and *CSF1R*
CBTS‐35	B	BM	Female	B‐ALL	*PAX5::JAK2*	*PAX5::JAK2*	/	/
CBTS‐28	B	BM	Male	B‐ALL	—	/	*NUP214::ABL1*	/
CBTS‐34	B	BM	Female	B‐ALL	—	—	*P2RY8::CRLF2*	/
CBTS‐31	B	PB	Female	Healthy	—	—	/	/
CBTS‐33	B	PB	Female	Healthy	—	—	/	/
CBTS‐36	C	BM	Male	B‐ALL	*EBF1::PDGFRB*	—	/	Possible rearrangement of *PDGFRB* and *CSF1R*
CBTS‐38	C	BM	Female	B‐ALL	*ENSG00000277856* *::EPOR*	*IGH::EPOR*	/	/
CBTS‐37	C	BM	Male	B‐ALL	—	/	/	Possible rearrangement of *CRLF2*
CBTS‐39	C	BM	Male	B‐ALL	—	/	/	Possible rearrangement of *CRLF2*

Abbreviations: B‐ALL: B‐cell acute lymphoblastic leukemia; BM: bone marrow; PB: peripheral blood; —: negative result; /: not tested.

Among the 26 samples in group A, 15 samples were from B‐ALL patients and 11 were from healthy controls. PACLseq achieved a sensitivity of 93.33% and a specificity rate of 100% for the positive samples, while the negative samples exhibited a specificity rate of 100%. One positive sample (CBTS‐15) was not detected. Subsequently, we repeated the RT‐PCR analysis on this sample, yielding a fusion frequency of 0.3%. The PACLseq results for the remaining samples exhibited complete concordance with the clinical findings. Among the nine samples in group B, seven tested positive and two tested negative. The positive samples in this group achieved a sensitivity of 71.43% with a sensibility rate of 100%, and the negative samples exhibited a specificity rate of 100%. Fusions were not detected in CBTS‐28 (*NUP214::ABL1*) and CBTS‐34 (*P2RY8::CRLF2*) in group B. In group C, PACLseq successfully detected an *EBF1::PDGFRB* fusion; however, the other three samples were not detected, likely due to noncoding fusions with significantly elevated target gene depth.

It is remarkable that we identified a new fusion, *ZBTB5::JAK2* in sample CBTS‐25 (Figure 3B), which was further substantiated through both RNA‐seq and Sanger sequencing. It is formed from exon 2 of *ZBTB5* gene (NM_014872.3) (chr9: 37441386:−) and exon 9 of *JAK2* gene (NM_004972.4) (chr9 5064881:+). After a comprehensive search across various databases, including the TICdb database, the cancer genome atlas program (TCGA) fusion gene database, the catalogue of somatic mutations in cancer (COSMIC) gene fusions database, ChimerDB 4.0 database [25], and Pubmed, we conclusively validated the novelty of the *ZBTB5::JAK2* fusion.

The median duration for fusion gene detection in the aforementioned Ph‐like ALL samples in the clinical samples was 14.5 days, with a corresponding median expense of 8000 CNY (1,100 $, Figure 3C, Table 2). In the blind test validation section of our study, the PACLseq method demonstrated a significantly reduced time and cost requirement, with a duration of 3 days and an expected price of 4000 CNY (550 $).

Furthermore, we conducted non‐targeted long‐read transcriptome sequencing on three available samples (CBTS‐36, CBTS‐4, and CBTS‐22) using QNome platform with clean bases ranging from 3265 to 4715 Mb. Among them, fusion genes were successfully detected in CBTS‐36 and CBTS‐4, but not in CBTS‐22. Notably, CBTS‐36 and CBTS‐4 exhibited 2483‐fold and 93‐fold of enrichment rate, respectively (Table 3). Besides, nanopore sequencing allows for the multiplexing of different samples on the same cell, reducing costs in clinical applications. To evaluate the multiplex performance on the QNome nanopore sequencing platform, we sequenced three samples that were indexed with different barcodes and pooled at equal molar concentrations. The analysis results remained consistent with the initial findings, confirming the reliability of multiplexing (Table S6).

**TABLE 3:** Fusion enrichment fold compared with non‐target RNAseq.

Sample ID	CBTS‐36		CBTS‐4		CBTS‐22	
	PACLseq	Non‐tageted RNAseq	PACLseq	Non‐tageted RNAseq	PACLseq	Non‐tageted RNAseq
QC bases (Mb)	350.05	3265	274.46	3490.75	499.75	4714.78
Number of fusion‐support‐reads	2662	10	249	34	164	0
Enrichment fold	2482.91		93.15		NA	

## 3 Discussion

We developed PACLseq, a novel method to detect fusion gene partners with a rapid turnaround time of 3 days, based on nanopore sequencing technology. Subsequently, we developed a panel specifically designed for the identification of Ph‐like ALL fusion genes and validated its diagnostic capability. This holds immense significance within the diagnostic and therapeutic workflow of Ph‐like ALL, addressing the critical need for early diagnosis and targeted drug intervention. Current clinical detection strategies for Ph‐like ALL fusion genes, aside from RNA‐seq, often require the combination of multiple detection methods. RNA‐seq, however, is not widely used in clinical settings due to its complexity and relatively high cost. Besides, the sensitivity of RNA‐seq in the detection of fusions is object to fusion gene expression levels and the commingling of normal tissue elements within the sample [26, 27]. In contrast, PACLseq, through its targeted approach, enhances the enrichment of targeted genes, thereby increasing the sensitivity in the detection of fusions, particularly for low‐frequency fusions, but also facilitates the identification of novel fusion events. Our results demonstrated its capacity to discover novel fusions (CBTS‐25), potentially expanding the panel of fusion genes associated with Ph‐like ALL. Furthermore, PACLseq offers several advantages for resource‐limited clinical settings, including a compact instrument footprint, relatively low data output requiring minimal storage and computational resources, and a streamlined workflow once standardized. Its low‐throughput design supports flexible, on‐demand testing without the need for batching, making it well‐suited for single‐institution use and enabling timely detection in newly diagnosed patients.

RNA is highly unstable and susceptible to degradation. Presently, commercial NGS facilities commonly specify a prerequisite of an RIN ≥ 7 for subsequent steps of library construction and analysis. Previous investigations on degraded RNA have revealed that the efficacy of detecting fusions is not only influenced by RNA degradation but also by the distance from the fusion breakpoint to the 3' end of the gene. Even a slight decrease in RIN makes fusions challenging to detect when the distance exceeds 1 kb [28]. This effect is particularly notable in clinical specimens, where limited sample storage conditions prevail in clinical settings, leading to general RNA degradation. However, our research demonstrates the remarkable tolerance of PACLseq toward sample quality. The method achieves a sensitivity of 93.33% in samples with RIN > 3. The only false negative observed could potentially be attributed to an extremely low fusion frequency (only 0.3% detected upon retesting). PACLseq demonstrates a notable sensitivity of 71.45% even in highly degraded specimens with RIN ≤ 3. In group B, fusions were not detected in CBTS‐28 (NUP214::ABL1) and CBTS‐34 (P2RY8::CRLF2). Quality control data for these samples showed that CBTS‐28 had an average coverage depth of 3195x for the ABL1 gene, with 96.46% of the area exceeding 500x, while CBTS‐34 had a coverage depth of 611x for the CRLF2 gene, with 70.35% exceeding 500x. Since no abnormalities were found in the quality control data for these two samples, the failure to detect fusions might be due to severe sample degradation or other unknown factors. Additionally, PACLseq requires a minimal input sample quantity of only 10 ng of RNA, thereby conserving sample material. This feature proves particularly beneficial for patients encountering challenges in obtaining bone marrow samples.

It is important to note that the fusion frequency calculated by PACLseq did not correspond with the RT‐PCR result observed in the clinical setting. Additionally, each repeated experiment exhibited a distinct fusion frequency, which can be attributed to two pivotal factors. First, there is a time gap between the RT‐PCR and PACLseq procedures, which may lead to RNA degradation and a decline in sample quality compared to fresh samples. Second, the fusion detection and calculation method employed in long‐read sequencing is inherently less precise than the clinical PCR method. As a result, PACLseq is more suitable for qualitative analysis rather than quantitative analysis. Based on our comprehensive findings, we propose that PACLseq is capable of qualitatively detecting fusions at a fusion frequency as low as a 10^−2^ level, which is competent for diagnostic purposes.

Constrained by the mRNA material, the detection of fusions within non‐coding intervals poses a significant challenge for PACLseq, as well as other mRNA‐based methods. Consequently, complex fusions formed by chromosomal translocation between the enhancer region of *IGH/IGK* and either *CRLF2* or *EPOR*, such as *IGH::CRLF2*, *IGH::EPOR*, and *IGK::EPOR*, might be missed. For example, the samples “CBTS‐37” and “CBTS‐39” exhibited clinical FISH results suggestive of a *CRLF2* rearrangement. PACLseq detected a significantly elevated expression of the *CRLF2* gene, a common characteristic associated with *CRLF2* gene rearrangements [29]. However, PACLseq did not provide information regarding the fusion partner and breakpoint. Although this indirectly suggests the presence of an *IGH::CRLF2* fusion, limitations in both sample size and quality prevented us from conducting additional experiments to verify our hypotheses. Moreover, part of *IGH::EPOR* and *IGK::EPOR* are formed by the insertion of *IGH/IGK* in the middle of *EPOR* exon8, which often introduces non‐templated nucleotides at breakpoints, potentially complicating sequence alignment. In the case of sample “CBTS‐38,” the clinical result indicated an *IGH::EPOR* fusion using multiple RT‐PCR. However, the result obtained through LongGF was *ENSG00000277856::EPOR*, where ENSG00000277856 represents a paralog of *IGHV3‐43*. We are planning to optimize the pipeline to solve these problems in the future. Cases that remain undetectable may require whole‐genome sequencing for precise determination of fusion genes.

We acknowledge that our study has certain limitation in panel size. First, PACLseq was designed to detect seven gene‐related fusion genes in Ph‐like ALL (*ABL2*, *CSF1R*, *PDGFRB*, *JAK2*, *ABL1*, *EPOR*, and *CRLF2*), with a detection capability encompassing 97.6% of cases with fusion genes [30]. Nonetheless, it lacks the capability to detect cases with fusions composed of other genes or those with mutations but without fusion genes, constituting approximately 15%–20% of Ph‐like ALL cases [31]. The definition and diagnosis of Ph‐like ALL still rely on gene expression profiles. While the identification of specific fusion genes can simplify and directly diagnose most cases, it cannot completely substitute for the comprehensive diagnosis of Ph‐like ALL by gene expression profiles. Second, due to the rarity of ABL2 fusion‐positive cases, we were unable to include ABL2 fusions in the blinded samples, so we cannot directly prove PACLSeq's ability to detect ABL2 fusions. But the average coverage depth of the ABL2 probe region in the blinded samples reached 1202x, with coverage across all target regions (Table S3, Figure S2). This demonstrates our ability to successfully capture ABL2 gene, and we are confident that if such fusion‐positive samples were included, PACLSeq would be capable of detecting them effectively.

## 4 Conclusion

In conclusion, we have successfully established a novel detection method for identifying Ph‐like ALL‐related fusion genes based on PACLseq. Through the validation of clinical samples, this method has demonstrated reliable detection results, along with a short turnaround time, cost‐effectiveness, a streamlined workflow, and resilience towards poor RNA quality and small sample input. Consequently, it proves to be an excellent choice for diagnosis of Ph‐like ALL in clinical settings. In the future, we intend to conduct prospective studies using large sample cohorts to further evaluate the reliability of PACLseq in diagnosing Ph‐like ALL, as well as explore the performance of long‐read sequencing for fusion gene detection. Additionally, we plan to design other panels to investigate further clinical applications of PACLseq.

## 5 Methods and Materials

### 5.1 Standard and Clinical Samples

Commercial *BCR::ABL1* standard sample was purchased as RNA format (*BCR::ABL1*, P210 Fusion, CBP20061R, Cobioer Biosciences Co., Ltd). Clinically confirmed positive validation samples were obtained from the peripheral blood or bone marrow, and the fusion frequency was quantified using RT‐PCR in the clinical setting. The clinical blind‐test negative samples were collected from the peripheral blood of healthy donors, while the clinical blind‐test positive samples were obtained from the bone marrow of first‐diagnosed B‐ALL patients. All healthy control samples included in this study underwent confirmatory multiplex RT‐PCR and were verified to be negative. The presence of Ph‐like fusion genes was clinically confirmed using FISH, multiplex RT‐PCR, or RNA‐seq methods performed in Kindstar Globalgene Technology, Inc. RNA extraction from both bone marrow and peripheral blood samples was performed using the TRIzol‐based RNA extraction method.

### 5.2 Commercial Probe Synthesis

In this study, we designed a detection panel associated with Ph‐like ALL, specifically *ABL2*, *CSF1R*, *PDGFRB*, *JAK2*, *ABL1*, *EPOR*, and *CRLF2* as target genes. The seven genes mentioned above collectively encompass 97.6% of the fusion genes associated with the Ph‐like ALL [30]. We designed probes to capture long fragments containing these genes. The transcript information for these genes was initially obtained from National Center for Biotechnology Information (NCBI) RefSeq. Subsequently, the exon regions of all transcripts were merged for each gene to establish a merged region for target capture (Table S2). Commercially synthesized probes were designed as biotinylated 100‐mers with a ∼3.6x tiling density against the merged region by iGeneTech (T040V19), and the target capture kit TargetSeq is also provided by iGeneTech (C10831).

### 5.3 Library Construction

The size distribution of mRNA was assessed using the Agilent Tapestation 4150 system, and samples with a RIN (RNA Integrity Number) value of ≤ 3 were determined to have undergone significant degradation. The mRNA samples were then chemically fragmented, followed by the synthesis of double‐stranded cDNA. Subsequently, the cDNA was utilized for library construction, which included pre‐capture library construction, target enrichment, and post‐capture library construction, according to the instructions provided by the manufacturer (mRNA‐seq Lib Prep Kit for Illumina, RK20302, ABclonal). For target enrichment, the pre‐capture library was subjected to capture using the iGeneTech TargetSeq One Hyb & Wash Kit with Eco Universal Blocking Oligo (iGeneTech, C10732) following the provided protocol. For more detailed information, please refer to the Supporting Information.

### 5.4 QNome‐3841 Nanopore Sequencing

After the post‐capture library construction, the library product was prepared for nanopore sequencing. Specifically, the product (300fmol) underwent end‐repair and nanopore adapter ligation using the QNome‐3841 library preparation protocol (QLK‐V1.1.1, QitanTech). Subsequently, the library product (80fmol) was loaded onto the flow cell and sequenced on QNome‐3841 nanopore sequencer (Figure S3). For Illumina short‐fragment sequencing, the libraries were sent to Novogene for sequencing on the Novaseq6000 platform. For more detailed information, please refer to the Supporting Information.

### 5.5 Bioinformatics Analysis

We have developed a streamlined bioinformatics workflow for analyzing QNome nanopore data, which can be found at https://github.com/HuanYuu/TargetFusion. This workflow incorporates quality control, reference alignment, fusion detection, and statistical analysis. The sequenced fastq files underwent filtering using nanofilt to remove reads with lengths shorter than 100 bp or quality scores lower than 7. The filtered reads were then aligned to the ensemble GRCh38 reference using minimap2 with the splice‐aware option (‐acx). Nanostat and mosdepth were used separately for subsequent calculations. To identify fusions, we evaluated several candidate software tools, including LongGF [20], JAFFAL [21], Genion [22], and Fusionseeker [23]. A *BCR::ABL1* standard and two clinical positive samples, totaling seven libraries, were utilized for performance evaluation (Supporting Information).

### 5.6 Data Sharing Statement

The QNome nanopore sequence datasets data generated in this study have been deposited in the NCBI sequence read archive under accession number PRJNA1002848. All data are also available from the authors upon reasonable request.

## Author Contributions

T. L. and D. X. conceived the project. H. Z. and T. L. provided patient samples and clinical data. H. Z. performed RNA extractions, and Y. C. and B. H. performed library preparation and targeted sequencing. H. Y., J. L., and K. J. performed the bioinformatic analysis. H. Z. and H. Y. wrote the manuscript with input from all authors. H. Z., H. Y., T. L., and D. X. finalized the manuscript. All authors revised and approved the final version of the manuscript.

## Ethics Statement

This study was approved by the Biomedical Ethics Committee of West China Hospital (approval number: 2023‐390).

## Conflicts of Interest

Huan Yu, Yanmei Chen, Kai Jiang, Beibei Huo, and Jialin Li are employees of Qitan Technology Ltd. They contributed to statistical analysis and manuscript preparation. However, the study design, data interpretation, and conclusions were determined independently by all authors, and the company had no influence on the scientific outcomes of the research. Other authors declare no conflicts of interest.

## Supporting information



Figure S1. The scheme of library construction.
Figure S2. The coverage of ABL2.
Figure S3. The QNome‐3841 Nanopore sequencer.
Table 1. Ph‐like fusion genes that have been reported
Table 2. The target capture region designed based on exons.
Table 3. Quality control metrics for tested samples.
Table 4. Fusion detected in the standard samples
Table 5. Fusion detection performance evaluation across 4 tools
Table 6. Fusion detection using PACLseq, and current clinical detection method turnaround time and cost for blinded samples

## Data Availability

The QNome nanopore sequence datasets generated in this study have been deposited in the NCBI sequence read archive under accession number PRJNA1002848. All data are also available from the authors upon reasonable request.
