# Supplementary material for: PACLseq: A Standalone Diagnostic Method for Ph‐Like Acute Lymphoblastic Leukemia Using Nanopore Sequencing
Source: MedComm (2020). 2025 Sep 16;6(10):e70360. doi: 10.1002/mco2.70360 (PMC12441304; doi:10.1002/mco2.70360)
Supplement: Supplementary file 1 — Figure S1. The scheme of library construction. Figure S2. The coverage of ABL2. Figure S3. The QNome‐3841 Nanopore sequencer. Table 1. Ph‐like fusion genes that have been reported Table 2. The target capture region designed based on exons. Table 3. Quality control metrics for tested samples. Table 4. Fusion detected in the standard samples Table 5. Fusion detection performance evaluation across 4 tools Table 6. Fusion detection using PACLseq, and current clinical detection method turnaround time and cost for blinded samples [file MCO2-6-e70360-s001.docx]

**PACLseq: A Standalone Diagnostic Method for Ph-like Acute Lymphoblastic Leukemia Using Nanopore Sequencing**

**Running Head:** Standalone fusion detection for Ph-like ALL

Hang Zhang^1,*^, Huan Yu^3,*^, Yanmei Chen^3^, Kai Jiang^3^, Beibei Huo^3^, Jialin Li^3^, Ting Liu^1^, Dan Xie^2^.

^1^ Department of Hematology, Institute of Hematology, West China Hospital of Sichuan University, Chengdu, Sichuan, 610041, China

^2^ Laboratory of Omics Technology and Bioinformatics, Frontiers Science Center for Disease-related Molecular Network, State Key Laboratory of Biotherapy, West China Hospital, Sichuan University, Chengdu, Sichuan, 610041, China

^3^ Qitan Technology Ltd., Chengdu, Sichuan, 610041, China

^*^ Hang Zhang and Huan Yu contributed equally to this work.

**Corresponding authors:**

Ting Liu

Department of Hematology, Institute of Hematology, West China Hospital of Sichuan University, No.37 Guoxue Alley, Wuhou District, Chengdu City, Sichuan Province, PR China

email: [liuting@scu.edu.cn](mailto:liuting@scu.edu.cn)

Dan Xie

Laboratory of Omics Technology and Bioinformatics, Frontiers Science Center for Disease-related Molecular Network, State Key Laboratory of Biotherapy, West China Hospital of Sichuan University, No.37 Guoxue Alley, Wuhou District, Chengdu City, Sichuan Province, PR China

email: [danxie@scu.edu.cn](mailto:danxie@scu.edu.cn)

**Supplementary Figures**


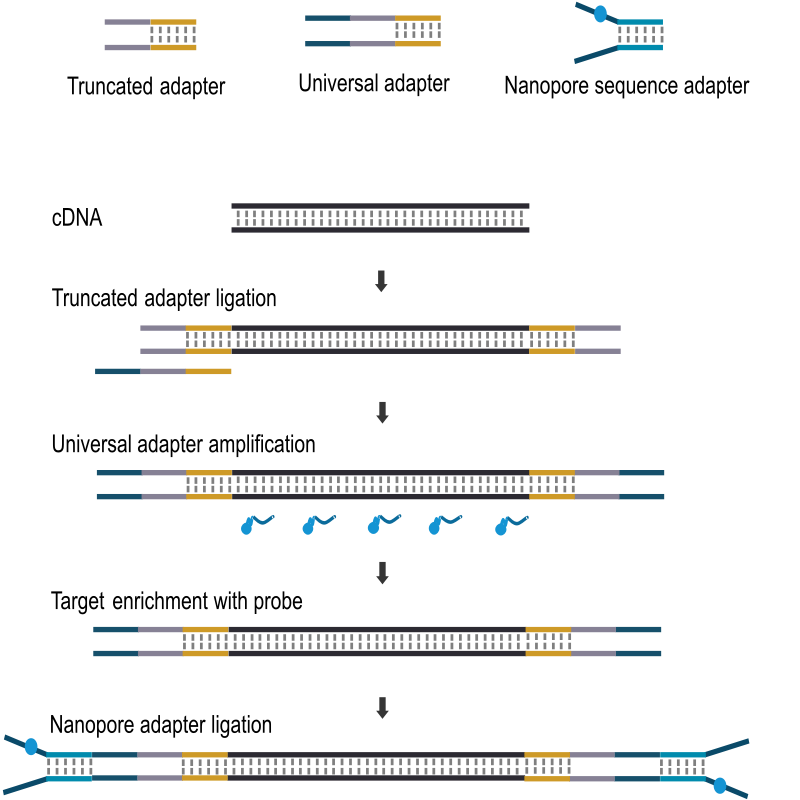


**Figure S1. The scheme of library construction.** The cDNA product is subjected to end-repair and then ligated with an RNA Truncated adapter. Following this, a Universal PCR adapter is ligated to the product, and the amplified cDNA is purified. Subsequently, the target genes are captured and ligated with the Nanopore adapter for further processing.

**
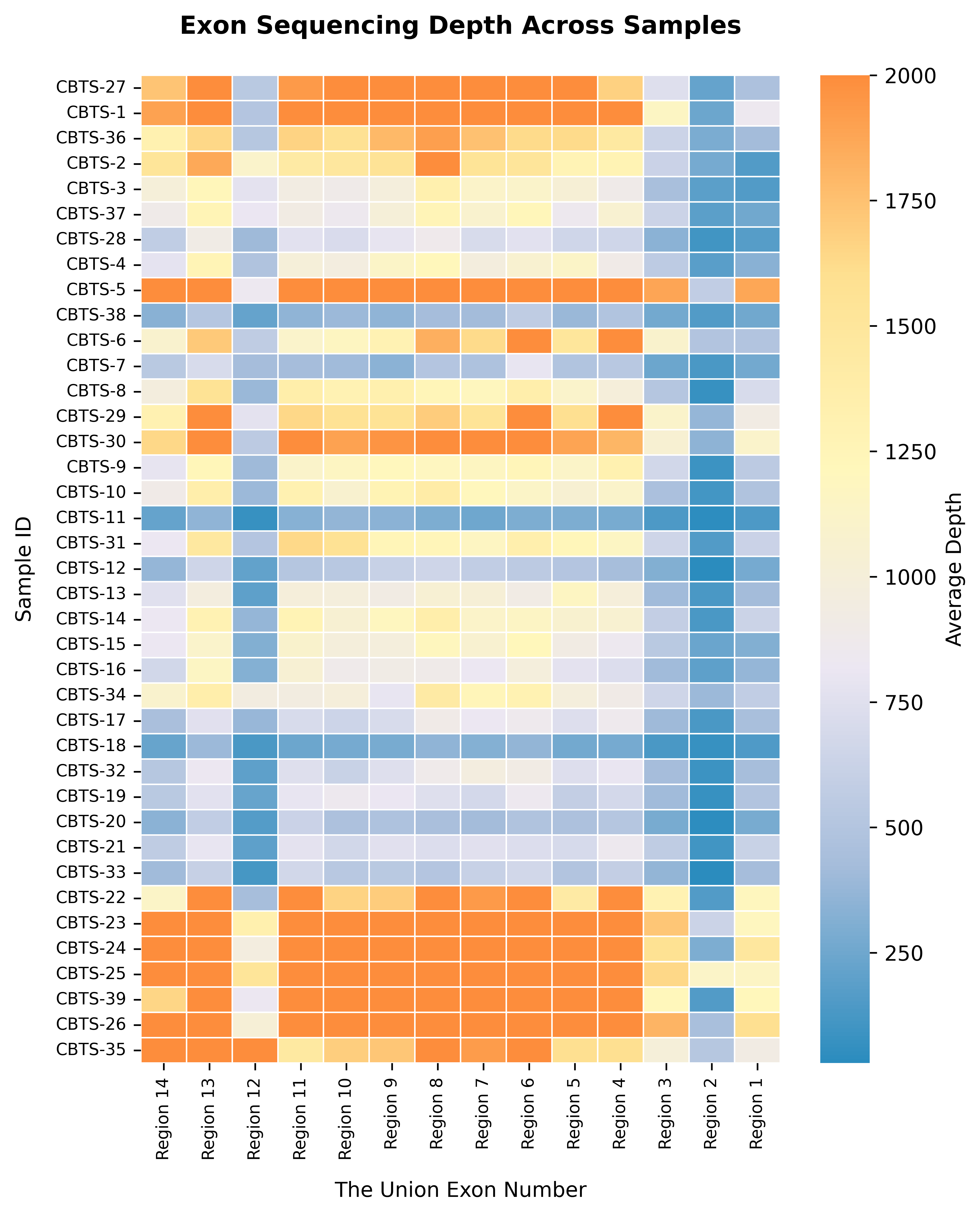
**

**Figure S2. The coverage of ABL2.** The average depth of the ABL2 gene probe design region (all exon regions combined) for blinded testing samples.


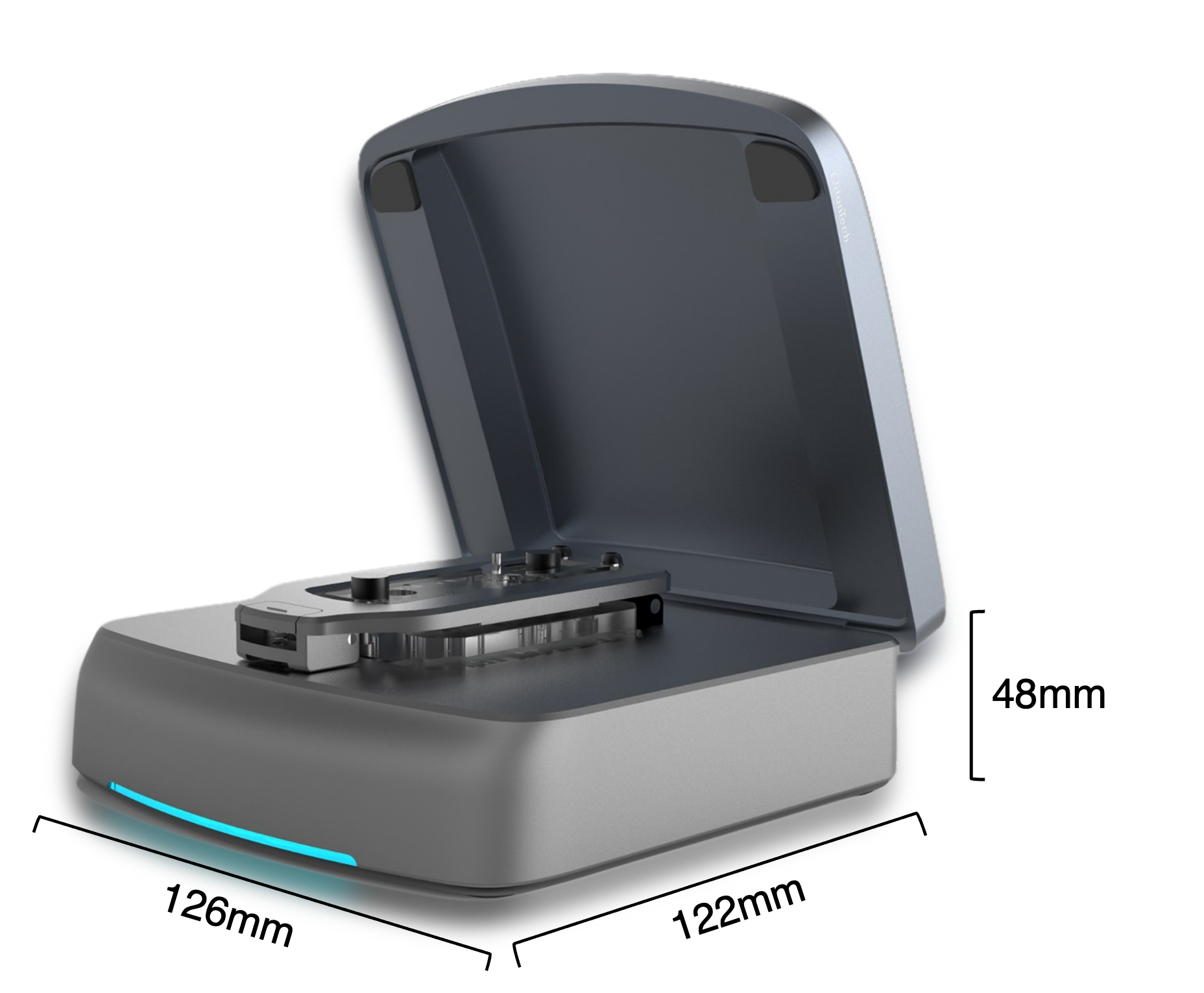


**Figure S3. The QNome-3841 Nanopore sequencer.**

**Supplementary Tables:**

**Supplementary Table 1. Ph-like fusion genes that have been reported**

| Kinase Gene | | Partner Gene |
| --- | --- | --- |
| *ABL* Family Kinases | |  |
|  | *ABL1* | *ETV6* ^1^, *NUP214* ^1,2^, *RCSD1* ^1,2^, *RANBP2* ^1^, *SNX2* ^1,2^, *ZMIZ1* ^1,3^, *SNX1* ^4^, *FOXP1* ^3^, *SFPQ* ^2^, *NUP153* ^5^, *LSM14A* ^5^, *CENPC* ^5^, *SPTAN1* ^6^, *INPP5D* ^7,8^, *MYO18B* ^9^, *CNTRL* ^10^ |
|  | *ABL2* | *PAG1* ^1,2^, *RCSD1* ^1,3^, *ZC3HAV1* ^1,2^ |
|  | *CSF1R* | *SSBP2* ^1^, *MEF2D* ^2^, *TBL1XR1* ^5^ |
|  | *PDGFRA* | *FIP1L1* ^2^ |
|  | *PDGFRB* | *EBF1* ^1-3^, *SSBP2* ^1^, *TNIP1* ^1^, *ZEB2* ^1^, *ETV6* ^5^, *ZMYND8* ^5^, *SNX29* ^2^, *ATF7IP* ^6^, *CCDC88C* ^11^, *SATB1* ^12^, *TERF2* ^13^, *GTF2I* ^14^ , *TPR* ^15^, *CD74* ^16,17^ |
|  | *LYN* | *GATAD2A* ^5^, *NCOR1* ^18^ |
| *JAK-STAT* Pathway | |  |
|  | *CRLF2* | *IGH* ^6^, *P2RY8* ^19^, *CSF2RA* ^20^ |
|  | *JAK2* | *ATF7IP* ^1,2^, *BCR* ^1^, *EBF1* ^1^, *ETV6* ^1,2^, *PAX5* ^2^, *SSBP2* ^1,2^, *STRN3* ^1^, *TERF2* ^1,12^, *TPR* ^1^, *ZNF430* ^2^, *SMU1* ^2^, *ZBTB46* ^2^, *USP25* ^5^, *GOLGA5* ^6^, *SNX29* ^2^, *RFX3* ^5^, *OFD1* ^18^, *STRBP* ^21^, *ZBTB20* ^22^, *KIAA1432* ^12^, *ZNF274* ^5,12^, *HMBOX1* ^23^, *PCM1* ^12^, *PPFIBP1* ^1,2^, *NIN* ^12^,*SPAG9* ^24^, *RNPC3* ^25^, *GOLGA4* ^26^, *GOLGB1* ^27^, *ZBTB44* ^27^ |
|  | *EPOR* | *IGH* ^1,12,28^, *IGK* ^1,28^, *LAIR1* ^28^, *THADA* ^2^ |
|  | *TSLP* | *IQGAP2* ^1^ |
|  | *TYK2* | *MYB* ^1^, *SMARCA4* ^2^, *ZBTB46* ^23^, *ZNF430* ^2^ |
|  | *FLT3* | *ZMYM2* ^2^ |
|  | *IL2RB* | *MYH9* ^1^ |
| Other Kinases | |  |
|  | *DGKH* | *ZFAND3* ^1^ |
|  | *NTRK3* | *ETV6* ^1,2^ |
|  | *PTK2B* | *KDM6A* ^1^, *STAG2* ^1^, *TMEM2* ^2^, *CEMIP2* ^29^ |
|  | *FGFR1* | *BCR* ^30^, *KIF5B* ^31^, *ZMYM2* ^32^, *HOOK3* ^29^ |
|  | *BLNK* | *DNTT* ^2^ |
|  | *CBL* | *KANK1* ^2^ |

A total of 95 fusion genes have been reported, 76.8% (73/95) of them were included in our panel.

**Supplementary Table 2. The target capture region designed based on exons.**

| #GeneName(GRCh38) | Chr | region_start | region_end | region_length |
| --- | --- | --- | --- | --- |
| ABL2 | chr1 | 179099330 | 179109441 | 10111 |
| ABL2 | chr1 | 179110281 | 179110455 | 174 |
| ABL2 | chr1 | 179110580 | 179110845 | 265 |
| ABL2 | chr1 | 179112308 | 179112398 | 90 |
| ABL2 | chr1 | 179114877 | 179115030 | 153 |
| ABL2 | chr1 | 179117331 | 179117516 | 185 |
| ABL2 | chr1 | 179118586 | 179118764 | 178 |
| ABL2 | chr1 | 179120189 | 179120274 | 85 |
| ABL2 | chr1 | 179121594 | 179121867 | 273 |
| ABL2 | chr1 | 179126376 | 179126672 | 296 |
| ABL2 | chr1 | 179131310 | 179131481 | 171 |
| ABL2 | chr1 | 179133311 | 179133374 | 63 |
| ABL2 | chr1 | 179142932 | 179143076 | 144 |
| ABL2 | chr1 | 179229240 | 179229684 | 444 |
| CSF1R | chr5 | 150053290 | 150054224 | 934 |
| CSF1R | chr5 | 150054321 | 150054430 | 109 |
| CSF1R | chr5 | 150055236 | 150055336 | 100 |
| CSF1R | chr5 | 150056025 | 150056137 | 112 |
| CSF1R | chr5 | 150056218 | 150056341 | 123 |
| CSF1R | chr5 | 150057286 | 150057384 | 98 |
| CSF1R | chr5 | 150057503 | 150057592 | 89 |
| CSF1R | chr5 | 150059699 | 150059862 | 163 |
| CSF1R | chr5 | 150060861 | 150060972 | 111 |
| CSF1R | chr5 | 150061490 | 150061595 | 105 |
| CSF1R | chr5 | 150061722 | 150061849 | 127 |
| CSF1R | chr5 | 150068214 | 150068330 | 116 |
| CSF1R | chr5 | 150069872 | 150070063 | 191 |
| CSF1R | chr5 | 150070181 | 150070302 | 121 |
| CSF1R | chr5 | 150070455 | 150070571 | 116 |
| CSF1R | chr5 | 150073300 | 150073493 | 193 |
| CSF1R | chr5 | 150077275 | 150077435 | 160 |
| CSF1R | chr5 | 150078111 | 150078248 | 137 |
| CSF1R | chr5 | 150080051 | 150080336 | 285 |
| CSF1R | chr5 | 150080766 | 150081024 | 258 |
| CSF1R | chr5 | 150086378 | 150086607 | 229 |
| CSF1R | chr5 | 150091456 | 150091561 | 105 |
| CSF1R | chr5 | 150092536 | 150092624 | 88 |
| CSF1R | chr5 | 150113260 | 150113372 | 112 |
| PDGFRB | chr5 | 150113838 | 150115946 | 2108 |
| PDGFRB | chr5 | 150117617 | 150117850 | 233 |
| PDGFRB | chr5 | 150118746 | 150118852 | 106 |
| PDGFRB | chr5 | 150119466 | 150119566 | 100 |
| PDGFRB | chr5 | 150120011 | 150120123 | 112 |
| PDGFRB | chr5 | 150120887 | 150121010 | 123 |
| PDGFRB | chr5 | 150121203 | 150121322 | 119 |
| PDGFRB | chr5 | 150121879 | 150122040 | 161 |
| PDGFRB | chr5 | 150123041 | 150123201 | 160 |
| PDGFRB | chr5 | 150124249 | 150124360 | 111 |
| PDGFRB | chr5 | 150124726 | 150124831 | 105 |
| PDGFRB | chr5 | 150125444 | 150125577 | 133 |
| PDGFRB | chr5 | 150126519 | 150126614 | 95 |
| PDGFRB | chr5 | 150129756 | 150129968 | 212 |
| PDGFRB | chr5 | 150130538 | 150130662 | 124 |
| PDGFRB | chr5 | 150131978 | 150132094 | 116 |
| PDGFRB | chr5 | 150132749 | 150132942 | 193 |
| PDGFRB | chr5 | 150133585 | 150133760 | 175 |
| PDGFRB | chr5 | 150133880 | 150134008 | 128 |
| PDGFRB | chr5 | 150134715 | 150135016 | 301 |
| PDGFRB | chr5 | 150135554 | 150135878 | 324 |
| PDGFRB | chr5 | 150137007 | 150137053 | 46 |
| PDGFRB | chr5 | 150155396 | 150155845 | 449 |
| JAK2 | chr9 | 4985085 | 4985630 | 545 |
| JAK2 | chr9 | 4985939 | 4986022 | 83 |
| JAK2 | chr9 | 5021962 | 5022213 | 251 |
| JAK2 | chr9 | 5029782 | 5029906 | 124 |
| JAK2 | chr9 | 5041692 | 5041907 | 215 |
| JAK2 | chr9 | 5044402 | 5044520 | 118 |
| JAK2 | chr9 | 5050685 | 5050831 | 146 |
| JAK2 | chr9 | 5054562 | 5054884 | 322 |
| JAK2 | chr9 | 5055668 | 5055788 | 120 |
| JAK2 | chr9 | 5064882 | 5065040 | 158 |
| JAK2 | chr9 | 5066677 | 5066789 | 112 |
| JAK2 | chr9 | 5069021 | 5069208 | 187 |
| JAK2 | chr9 | 5069924 | 5070052 | 128 |
| JAK2 | chr9 | 5072491 | 5072626 | 135 |
| JAK2 | chr9 | 5073697 | 5073785 | 88 |
| JAK2 | chr9 | 5077452 | 5077580 | 128 |
| JAK2 | chr9 | 5078305 | 5078444 | 139 |
| JAK2 | chr9 | 5080228 | 5080380 | 152 |
| JAK2 | chr9 | 5080532 | 5080683 | 151 |
| JAK2 | chr9 | 5081724 | 5081861 | 137 |
| JAK2 | chr9 | 5089673 | 5089863 | 190 |
| JAK2 | chr9 | 5090445 | 5090570 | 125 |
| JAK2 | chr9 | 5090738 | 5090911 | 173 |
| JAK2 | chr9 | 5123003 | 5123121 | 118 |
| JAK2 | chr9 | 5126332 | 5126446 | 114 |
| JAK2 | chr9 | 5126683 | 5129944 | 3261 |
| ABL1 | chr9 | 130713880 | 130714455 | 575 |
| ABL1 | chr9 | 130835253 | 130835525 | 272 |
| ABL1 | chr9 | 130854063 | 130854237 | 174 |
| ABL1 | chr9 | 130854800 | 130855096 | 296 |
| ABL1 | chr9 | 130862762 | 130863035 | 273 |
| ABL1 | chr9 | 130872128 | 130872213 | 85 |
| ABL1 | chr9 | 130872859 | 130873037 | 178 |
| ABL1 | chr9 | 130874867 | 130875052 | 185 |
| ABL1 | chr9 | 130878414 | 130878567 | 153 |
| ABL1 | chr9 | 130880067 | 130880157 | 90 |
| ABL1 | chr9 | 130880499 | 130880664 | 165 |
| ABL1 | chr9 | 130883968 | 130887675 | 3707 |
| EPOR | chr19 | 11377206 | 11378595 | 1389 |
| EPOR | chr19 | 11378690 | 11378778 | 88 |
| EPOR | chr19 | 11380883 | 11380971 | 88 |
| EPOR | chr19 | 11381055 | 11381209 | 154 |
| EPOR | chr19 | 11381691 | 11381849 | 158 |
| EPOR | chr19 | 11381929 | 11382105 | 176 |
| EPOR | chr19 | 11383096 | 11383232 | 136 |
| EPOR | chr19 | 11384092 | 11384314 | 222 |
| CRLF2 | chrX | 1190489 | 1191160 | 671 |
| CRLF2 | chrX | 1193217 | 1193302 | 85 |
| CRLF2 | chrX | 1196779 | 1196900 | 121 |
| CRLF2 | chrX | 1198561 | 1198724 | 163 |
| CRLF2 | chrX | 1202401 | 1202535 | 134 |
| CRLF2 | chrX | 1206432 | 1206599 | 167 |
| CRLF2 | chrX | 1208805 | 1208908 | 103 |
| CRLF2 | chrX | 1212555 | 1212723 | 168 |
| CRLF2 | chrY | 1190489 | 1191160 | 671 |
| CRLF2 | chrY | 1193217 | 1193302 | 85 |
| CRLF2 | chrY | 1196779 | 1196900 | 121 |
| CRLF2 | chrY | 1198561 | 1198724 | 163 |
| CRLF2 | chrY | 1202401 | 1202535 | 134 |
| CRLF2 | chrY | 1206432 | 1206599 | 167 |
| CRLF2 | chrY | 1208805 | 1208908 | 103 |
| CRLF2 | chrY | 1212555 | 1212723 | 168 |

**Supplementary Table 3. Quality control metrics for tested samples.**

| SampleID | Raw bases(Mb) | Raw reads(M) | QC bases(Mb) | QC reads(M) | QC read length N50 | QC mean read quality | QC pass rate of reads(%) | Mapping rate of reads(%) | Capture rate of bases (%) | Average depth on target (x) | Coverage ≥ 1x (%) | Coverage ≥ 500x (%) | ABL2 average depth (x) | ABL2 covreage ≥1x (%) | ABL2 covreage ≥500X (%) | CSF1R average depth (x) | CSF1R covreage ≥1x (%) | CSF1R covreage ≥500X (%) | PDGFRB average depth (x) | PDGFRB covreage ≥1x (%) | PDGFRB covreage ≥500X (%) | JAK2 average depth (x) | JAK2 covreage ≥1x (%) | JAK2 covreage ≥500X (%) | ABL1 average depth (x) | ABL1 covreage ≥1x (%) | ABL1 covreage ≥500X (%) | EPOR average depth (x) | EPOR covreage ≥1x (%) | EPOR covreage ≥500X (%) | CRLF2 average depth (x) | CRLF2 covreage ≥1x (%) | CRLF2 covreage ≥500X (%) |
| --- | --- | --- | --- | --- | --- | --- | --- | --- | --- | --- | --- | --- | --- | --- | --- | --- | --- | --- | --- | --- | --- | --- | --- | --- | --- | --- | --- | --- | --- | --- | --- | --- | --- |
| BCR-ABL1-NGS | 299.78 | 2 | 231.79 | 1.98 | 145 | 30.5 | 98.93 | 98.93 | 72.56 | 3922.25 | 87.67 | 60.16 | 2368.95 | 99.87 | 91.73 | 58.87 | 93.26 | 0.12 | 2.51 | 49.18 | 0 | 2302.56 | 100 | 71.6 | 23095.08 | 98.83 | 95.35 | 2524.75 | 100 | 97.35 | 18.97 | 84.31 | 0 |
| BCR-ABL1-NGS | 299.82 | 2 | 233.71 | 1.98 | 145 | 30.4 | 98.94 | 99.12 | 72.74 | 3972.96 | 87.77 | 60.6 | 2427.37 | 99.94 | 92.4 | 70.38 | 96.34 | 1.46 | 2.4 | 46.18 | 0 | 2263.27 | 100 | 72.25 | 23226.94 | 98.49 | 95.35 | 2550.21 | 100 | 97.22 | 13.45 | 90.51 | 0 |
| BCR-ABL1-QNome-short | 449.76 | 1.25 | 258.85 | 1.04 | 267 | 8.7 | 83.41 | 53.87 | 70.32 | 1862.96 | 87.91 | 56.8 | 1064.32 | 99.87 | 88.69 | 29.76 | 94.14 | 0 | 1.75 | 50.19 | 0 | 1154.94 | 100 | 60.74 | 9581.19 | 98.54 | 95.3 | 1320.13 | 100 | 88.97 | 9.79 | 85.67 | 0 |
| BCR-ABL1-QNome-short | 641.56 | 1.84 | 318.47 | 1.43 | 237 | 9 | 77.62 | 59.17 | 72.26 | 2652.45 | 88.38 | 58.14 | 1542.22 | 99.93 | 90.41 | 46.87 | 97.27 | 0 | 1.42 | 48.15 | 0 | 1534.09 | 100 | 63.77 | 14537.47 | 98.8 | 95.43 | 1900.61 | 100 | 93.49 | 12.22 | 95.53 | 0 |
| BCR-ABL1-QNome-long | 931.21 | 1.33 | 635.08 | 1.11 | 735 | 7.8 | 83.43 | 63.76 | 65.66 | 4239.7 | 90.83 | 61.09 | 1794.15 | 100 | 93.56 | 62.71 | 94.31 | 0 | 1.86 | 68.07 | 0 | 1773.9 | 100 | 73.61 | 20759.72 | 98.62 | 95.73 | 4178.74 | 100 | 96.93 | 11.57 | 96.03 | 0 |
| CCPVS-1 | 409.41 | 0.95 | 288.02 | 0.86 | 332 | 7.9 | 90.86 | 59.84 | 47.23 | 1370.14 | 95.4 | 57.39 | 394.8 | 100 | 31.9 | 1405.87 | 100 | 90.22 | 2137.9 | 100 | 92.48 | 4817.06 | 100 | 92.8 | 495.26 | 98.39 | 41.09 | 465.22 | 100 | 59.98 | 3.75 | 87.03 | 0 |
| CCPVS-2 | 450.54 | 1.04 | 308.24 | 0.95 | 318 | 8.2 | 91.72 | 61.84 | 66.41 | 2165.1 | 95.58 | 67.09 | 706.34 | 99.94 | 71.68 | 1605.09 | 95.38 | 90.48 | 183.11 | 100 | 0 | 10579.9 | 100 | 98.3 | 2095.56 | 99.45 | 93.73 | 1468.73 | 100 | 87.76 | 27.08 | 100 | 0 |
| CCPVS-1 | 1133.79 | 1.5 | 373.62 | 0.97 | 465 | 10.2 | 65.07 | 94.97 | 70.38 | 5032.5 | 96.1 | 87.74 | 1438.03 | 100 | 91.8 | 7102.95 | 100 | 91.7 | 11695.05 | 100 | 99.74 | 14027.21 | 100 | 100 | 2029.8 | 99.74 | 94.31 | 2977.46 | 100 | 93.9 | 25.69 | 100 | 0 |
| CCPVS-2 | 1143.2 | 1.47 | 526.06 | 1.06 | 665 | 8.9 | 72 | 88.29 | 53 | 4210.68 | 95.93 | 82.79 | 1376.8 | 100 | 91.49 | 4179.08 | 100 | 91.97 | 506.95 | 100 | 60.55 | 18636.79 | 100 | 100 | 4582.05 | 98.59 | 96.13 | 4971.83 | 100 | 97.88 | 64.63 | 100 | 0 |
| CCPVS-2 | 761.36 | 1.2 | 402.21 | 0.95 | 522 | 9 | 78.75 | 89.55 | 51.36 | 3312.73 | 95.92 | 74.94 | 1060.43 | 100 | 89.8 | 3139.8 | 99.98 | 91.99 | 386.97 | 100 | 7.85 | 15001.6 | 100 | 99.92 | 3426.53 | 98.54 | 95.6 | 3536.81 | 100 | 97.76 | 44.75 | 100 | 0 |
| CCPVS-2 | 715.96 | 1.21 | 443.39 | 1.04 | 498 | 8.6 | 85.97 | 88.02 | 60.35 | 4262.98 | 95.86 | 81.65 | 1413.31 | 100 | 92.26 | 3957.88 | 99.23 | 92.23 | 480.48 | 100 | 50.16 | 19623.56 | 100 | 99.97 | 4359.97 | 98.6 | 96.12 | 4223.12 | 100 | 98.55 | 64.73 | 100 | 0 |
| CCPVS-2 | 999.61 | 1.76 | 569.51 | 1.48 | 457 | 8.9 | 83.69 | 87.05 | 39.6 | 3624.58 | 95.9 | 79.13 | 1168.28 | 100 | 91.65 | 3650.46 | 99.57 | 92.11 | 453.78 | 100 | 34.16 | 15770.34 | 100 | 99.37 | 4041.78 | 98.7 | 95.94 | 4474.76 | 100 | 98.59 | 45.67 | 100 | 0 |
| CCPVS-2 | 978.93 | 1.78 | 547 | 1.51 | 409 | 9.5 | 84.66 | 91.55 | 67.49 | 6701.27 | 95.94 | 89.41 | 2317.85 | 100 | 95.99 | 7002.29 | 100 | 92.52 | 899.1 | 100 | 96.53 | 28749.13 | 100 | 100 | 7813.2 | 98.65 | 97.07 | 8917.93 | 100 | 100 | 90.3 | 100 | 0 |
| CCPVS-2 | 1266.62 | 2.35 | 606.95 | 1.94 | 349 | 10.7 | 82.51 | 95.42 | 48.05 | 6099.27 | 95.92 | 88.97 | 2033.77 | 100 | 95.69 | 5737.83 | 100 | 92.2 | 743.04 | 100 | 94.26 | 27344.23 | 100 | 100 | 7673.93 | 98.49 | 97.19 | 7994.13 | 100 | 99.71 | 74.12 | 100 | 0 |
| CCPVS-2 | 648.7 | 1.3 | 331.66 | 1.09 | 332 | 9.4 | 83.78 | 88.72 | 37.28 | 2226.05 | 95.22 | 69.66 | 771.97 | 100 | 77.32 | 2340.16 | 93.78 | 90.87 | 294.36 | 100 | 0 | 9741.22 | 100 | 98.88 | 2611.09 | 98.33 | 95.3 | 2790.5 | 100 | 96.27 | 39.46 | 98.82 | 0 |
| CCPVS-2 | 908.3 | 1.54 | 534.89 | 1.29 | 502 | 8.5 | 83.67 | 83.44 | 51.5 | 4098.62 | 95.84 | 80.32 | 1329.93 | 100 | 91.09 | 3543.61 | 99.35 | 91.97 | 479.18 | 100 | 44.21 | 18979.28 | 100 | 100 | 4277.01 | 98.37 | 95.45 | 4121.92 | 100 | 97.76 | 65.62 | 100 | 0 |
| CCPVS-2 | 690.18 | 1.39 | 422.11 | 1.21 | 396 | 8.4 | 87.1 | 80.4 | 30.79 | 1977.64 | 95.63 | 67.84 | 617.54 | 99.99 | 72.99 | 1702.53 | 97.42 | 90.46 | 220.61 | 100 | 0 | 9350.91 | 100 | 97.41 | 2029.79 | 98.31 | 94.73 | 1770.69 | 100 | 93.99 | 30.94 | 100 | 0 |
| CCPVS-2 | 613.33 | 1.25 | 352.26 | 1.09 | 357 | 8.8 | 87.28 | 87.04 | 37.06 | 2300.93 | 95.48 | 69.85 | 734.75 | 100 | 78.17 | 2176.3 | 95.86 | 90.72 | 269.76 | 100 | 0 | 10473.32 | 100 | 98.98 | 2558.52 | 98.33 | 94.78 | 2496.1 | 100 | 96.27 | 46.05 | 100 | 0 |
| CCPVS-2 | 567.08 | 1.16 | 365.45 | 1.04 | 386 | 8.5 | 89.47 | 81.19 | 39.99 | 2275.37 | 95.84 | 70.68 | 800.35 | 100 | 80.67 | 2232.27 | 99.28 | 91.08 | 313.15 | 100 | 0.19 | 10204.39 | 100 | 99.34 | 2512.4 | 98.47 | 94.75 | 2287.12 | 100 | 95.44 | 46.89 | 100 | 0 |
| CCPVS-3 | 402.95 | 0.45 | 163.89 | 0.3 | 804 | 8.6 | 66.09 | 89.19 | 36.44 | 848.75 | 95.65 | 54.21 | 475.78 | 100 | 48.12 | 1901.16 | 97.37 | 89.91 | 148.35 | 100 | 0 | 2811.64 | 100 | 85.38 | 833.8 | 98.5 | 89.45 | 401.58 | 100 | 39.73 | 23.01 | 99.94 | 0 |
| CCPVS-4 | 316.87 | 0.34 | 167.82 | 0.25 | 1,001.00 | 8.4 | 75.07 | 90.9 | 68.96 | 1575.35 | 96.06 | 65.44 | 652.1 | 100 | 66 | 2097.85 | 99.98 | 90.1 | 92.5 | 100 | 0 | 7230.21 | 100 | 98.3 | 1140.61 | 99.45 | 93.19 | 1514.87 | 100 | 91 | 18.73 | 100 | 0 |
| CCPVS-5 | 791.41 | 0.76 | 330.42 | 0.49 | 1,046.00 | 8.6 | 64.38 | 87.33 | 29.92 | 1212.05 | 95.89 | 57.89 | 462.55 | 100 | 45.2 | 2994.25 | 99.88 | 90.03 | 169.53 | 100 | 0 | 4433.59 | 100 | 95.5 | 847.4 | 98.41 | 89.84 | 992.92 | 100 | 86.4 | 31.68 | 100 | 0 |
| CCPVS-3 | 521.67 | 0.87 | 292.66 | 0.72 | 486 | 8.3 | 81.81 | 88.03 | 21.67 | 1072.86 | 95.62 | 64.62 | 618.4 | 99.83 | 76.39 | 2266.99 | 97.82 | 90.05 | 175.9 | 100 | 0 | 3657.7 | 100 | 89.97 | 1198.99 | 98.31 | 93.55 | 468.72 | 100 | 46.99 | 36.32 | 100 | 0 |
| CCPVS-6 | 1301.21 | 2.29 | 791.92 | 2 | 457 | 8.9 | 87.35 | 89.92 | 35.45 | 4999.54 | 95.68 | 89.59 | 2560.93 | 100 | 97.36 | 13908.25 | 97.9 | 91.42 | 1871.07 | 100 | 99.22 | 14643.53 | 100 | 99.97 | 4339.65 | 98.41 | 95.68 | 2542.4 | 100 | 95.06 | 39.05 | 99.75 | 0 |
| CCPVS-4 | 619.07 | 0.91 | 326.53 | 0.72 | 555 | 8.7 | 79.12 | 92.13 | 59.24 | 3383.46 | 95.74 | 75.13 | 1535.57 | 100 | 94.51 | 5173.48 | 97.9 | 90.94 | 257.68 | 100 | 0 | 14288.26 | 100 | 98.98 | 3241.18 | 98.75 | 95.9 | 3948.15 | 100 | 98.96 | 49.52 | 100 | 0 |
| CCPVS-7 | 731.2 | 1.12 | 363.4 | 0.86 | 519 | 8.8 | 76.91 | 89.2 | 44.11 | 2685.54 | 95.7 | 73.07 | 933.72 | 99.97 | 87.96 | 3282.27 | 97.8 | 90.89 | 152.01 | 100 | 0 | 11985.96 | 100 | 99.37 | 2563.93 | 98.57 | 95.06 | 3881.88 | 100 | 98.67 | 32.59 | 100 | 0 |
| CCPVS-5 | 529.76 | 0.79 | 336.96 | 0.67 | 614 | 8.4 | 84.87 | 87.31 | 43.39 | 2293.53 | 95.83 | 72.89 | 879.95 | 99.86 | 90.22 | 4992.59 | 100 | 90.94 | 287.84 | 99.98 | 3.35 | 8983.05 | 100 | 95.11 | 1781.66 | 98.21 | 94.2 | 1670.22 | 100 | 90.79 | 84.74 | 100 | 0 |
| CCPVS-8 | 842.84 | 1.45 | 504.48 | 1.24 | 470 | 8.9 | 85.78 | 90.43 | 40.78 | 3613.94 | 95.66 | 86.05 | 892.67 | 99.97 | 90.03 | 9910.61 | 97.85 | 91.3 | 663.14 | 100 | 92.05 | 15131.02 | 100 | 100 | 1965.24 | 98.24 | 94.73 | 1475.73 | 100 | 91.79 | 45.05 | 100 | 0 |
| CCPVS-3 | 461.2 | 0.61 | 266.92 | 0.47 | 753 | 8.2 | 77.72 | 80.53 | 48.3 | 1485.66 | 95.43 | 69.36 | 812.85 | 100 | 84.63 | 2891.92 | 94.93 | 90.32 | 199.39 | 100 | 0 | 5268.21 | 100 | 95.98 | 1310.5 | 98.6 | 93.73 | 608.51 | 100 | 66.57 | 48.7 | 100 | 0 |
| CCPVS-6 | 525.86 | 0.99 | 225.82 | 0.76 | 330 | 9.6 | 76.41 | 88.66 | 50.53 | 1991.35 | 95.43 | 82.99 | 1021.68 | 100 | 86 | 6064.75 | 95.38 | 91.06 | 740.33 | 100 | 85.26 | 5561.73 | 100 | 97.04 | 1475.42 | 98.37 | 93.73 | 1098.18 | 100 | 88.14 | 17.07 | 99.81 | 0 |
| CCPVS-4 | 609.47 | 1.33 | 313.11 | 1.12 | 303 | 8.9 | 83.78 | 80.62 | 15.19 | 723.62 | 95.16 | 36.08 | 311.78 | 100 | 5.2 | 954.49 | 95.17 | 88.71 | 39.23 | 99.74 | 0 | 3461.8 | 100 | 83.89 | 502.48 | 98.34 | 51.41 | 644.44 | 100 | 54.38 | 7.67 | 94.42 | 0 |
| CCPVS-7 | 780.84 | 1.62 | 428.21 | 1.37 | 348 | 8.6 | 84.2 | 78.48 | 20.89 | 1325.86 | 95.41 | 59.25 | 455.41 | 99.9 | 46.41 | 1653.64 | 95.24 | 90.32 | 70.71 | 100 | 0 | 5923.89 | 100 | 96 | 1091.12 | 98.5 | 93.24 | 1759.04 | 100 | 92.95 | 18.58 | 100 | 0 |
| CCPVS-5 | 815.77 | 1.68 | 416.24 | 1.39 | 333 | 9.2 | 82.43 | 84.52 | 37.71 | 2525.53 | 95.52 | 73.64 | 963.06 | 99.91 | 84.55 | 6944 | 96.25 | 91.25 | 401.13 | 100 | 17.61 | 8791.89 | 100 | 97.45 | 1777.88 | 98.57 | 93.87 | 2329.63 | 100 | 92.74 | 70.13 | 100 | 0 |
| CCPVS-8 | 1122.2 | 2.23 | 610.39 | 1.88 | 367 | 8.9 | 84.21 | 85.05 | 58.8 | 5819.61 | 95.7 | 87.82 | 1415.5 | 100 | 92.96 | 16719.65 | 97.85 | 91.68 | 980.16 | 100 | 97.23 | 23294.95 | 100 | 100 | 2712.11 | 98.49 | 94.46 | 2218.11 | 100 | 94.9 | 84.8 | 100 | 0 |
| CBTS-1 | 791.78 | 1.57 | 421.18 | 1.37 | 349 | 10.5 | 87.16 | 95.29 | 35.16 | 3134.66 | 95.85 | 83.91 | 2062.54 | 99.84 | 95.68 | 475.46 | 99.83 | 47.66 | 633.09 | 100 | 82.53 | 3377 | 100 | 91.87 | 6249.07 | 98.46 | 97.19 | 832.69 | 100 | 75.36 | 30532.41 | 100 | 100 |
| CBTS-2 | 582.6 | 0.91 | 409.45 | 0.83 | 632 | 9.6 | 91.16 | 91.64 | 18.12 | 1453.78 | 95.81 | 85.94 | 1262.64 | 99.69 | 93.55 | 945.28 | 100 | 87.33 | 532.88 | 100 | 73.72 | 3134.36 | 100 | 91.25 | 1875.87 | 98.39 | 94.08 | 2321.59 | 100 | 93.7 | 725.56 | 100 | 85.11 |
| CBTS-3 | 576.47 | 0.99 | 361.63 | 0.87 | 503 | 9.3 | 88.07 | 91.26 | 22.88 | 1625.88 | 95.84 | 84.75 | 861.65 | 99.81 | 92.05 | 1073.87 | 100 | 85.89 | 2216.59 | 100 | 98.95 | 1449.05 | 100 | 86.16 | 2801.77 | 98.37 | 96.33 | 321.5 | 100 | 23.35 | 8818.18 | 100 | 99.94 |
| CBTS-4 | 426.7 | 1.01 | 274.46 | 0.92 | 319 | 9.1 | 91.29 | 73.38 | 31.28 | 1190.51 | 94.91 | 63.39 | 856.35 | 99.86 | 82.41 | 3296.7 | 94.76 | 90.03 | 10.25 | 96.72 | 0 | 2285.44 | 100 | 85.42 | 1972.57 | 98.54 | 94.47 | 230.28 | 100 | 5.89 | 224.34 | 100 | 0 |
| CBTS-5 | 741.01 | 1.46 | 490.57 | 1.29 | 448 | 8.4 | 87.99 | 70.02 | 50.25 | 3191.08 | 95.35 | 79.87 | 3574.51 | 100 | 98.08 | 3145.88 | 93.95 | 91.97 | 75.45 | 99.98 | 0 | 5672.88 | 100 | 98.58 | 6533.49 | 98.75 | 97.16 | 981.3 | 100 | 91.87 | 2982.48 | 100 | 99.75 |
| CBTS-6 | 540.58 | 0.84 | 356.92 | 0.73 | 693 | 9.5 | 87.35 | 82.76 | 30.78 | 1974.77 | 95.93 | 87.05 | 1303.45 | 100 | 96.22 | 1427.74 | 100 | 95.29 | 936.39 | 100 | 95.73 | 1255.36 | 100 | 65.82 | 6137.4 | 98.59 | 97.92 | 4287.71 | 100 | 100 | 843.16 | 100 | 86.72 |
| CBTS-7 | 469.72 | 0.97 | 298.52 | 0.86 | 420 | 9.2 | 88.53 | 76.19 | 21.94 | 1064.41 | 96 | 56.79 | 447.75 | 100 | 41.34 | 1767.87 | 100 | 90.46 | 349.24 | 100 | 18.4 | 1530.01 | 100 | 69.51 | 1985.1 | 99.02 | 95.79 | 3138.37 | 100 | 97.64 | 224.19 | 100 | 15.26 |
| CBTS-8 | 856.39 | 1.93 | 573.65 | 1.78 | 349 | 8.9 | 92.14 | 74.13 | 16.87 | 1408.55 | 95.93 | 71.82 | 1009.3 | 100 | 90.33 | 384.17 | 100 | 35.6 | 746.23 | 100 | 93.97 | 1010.42 | 100 | 55.61 | 3132.78 | 98.6 | 96.36 | 263.29 | 100 | 3.57 | 10309.96 | 100 | 96.9 |
| CBTS-9 | 1064.76 | 2.46 | 597.39 | 2.12 | 307 | 9.9 | 86.47 | 83.95 | 17.61 | 1766.49 | 95.91 | 70.11 | 947.15 | 100 | 83.07 | 3420.55 | 100 | 90.34 | 251.93 | 100 | 0.82 | 6821.04 | 100 | 95.05 | 1761.87 | 98.44 | 93.76 | 1184.83 | 100 | 88.55 | 83.27 | 100 | 0 |
| CBTS-10 | 503.03 | 0.93 | 320.8 | 0.79 | 487 | 8.9 | 84.98 | 77.92 | 40.66 | 1800.18 | 95.17 | 78 | 948.04 | 100 | 85.8 | 4566.98 | 97.75 | 90.51 | 618.75 | 100 | 78.43 | 5859.61 | 100 | 95.39 | 1611.51 | 98.29 | 93.6 | 280.42 | 100 | 25.47 | 9.74 | 87.22 | 0 |
| CBTS-11 | 588.49 | 1.43 | 368.82 | 1.3 | 306 | 9.4 | 90.95 | 78.32 | 14.79 | 838.11 | 95.43 | 42.01 | 244.36 | 100 | 0 | 1272.92 | 95.24 | 89.93 | 86.97 | 100 | 0 | 3840.95 | 100 | 93.95 | 627.1 | 98.54 | 77.12 | 1116.69 | 100 | 85.57 | 7.69 | 99.44 | 0 |
| CBTS-12 | 505.97 | 1.2 | 314.93 | 1.07 | 316 | 9.2 | 89.44 | 78.11 | 21.74 | 1031.78 | 95.24 | 48.03 | 444.01 | 99.99 | 25.68 | 2306.04 | 95.38 | 90.15 | 152.26 | 100 | 0 | 4269.96 | 100 | 90.22 | 715.45 | 98.5 | 76.16 | 705.05 | 100 | 68.93 | 6.61 | 94.54 | 0 |
| CBTS-13 | 587.45 | 1.2 | 389.66 | 1.07 | 420 | 8.7 | 89.4 | 74.65 | 56.48 | 3071.9 | 95.59 | 72.18 | 780.08 | 99.97 | 84.56 | 3007.18 | 94.88 | 90.77 | 133.72 | 100 | 0 | 15388.93 | 100 | 99.99 | 2656.09 | 99.89 | 95.4 | 3668.56 | 100 | 98.42 | 56.64 | 99.81 | 0 |
| CBTS-14 | 909.89 | 2.05 | 596.77 | 1.88 | 349 | 9 | 91.86 | 77.14 | 21.93 | 1935.9 | 95.68 | 71.88 | 936.69 | 100 | 87.13 | 4462.4 | 97.35 | 90.77 | 319.86 | 100 | 0.84 | 7002.62 | 100 | 97.37 | 1758.74 | 98.67 | 93.94 | 1400.08 | 100 | 89.63 | 55.09 | 100 | 0 |
| CBTS-15 | 759.74 | 1.6 | 513.05 | 1.45 | 405 | 8.6 | 90.71 | 74.25 | 19.86 | 1608.17 | 95.9 | 76.5 | 829.61 | 99.91 | 90.54 | 2120.43 | 100 | 90.96 | 345.54 | 100 | 28.41 | 6259.42 | 100 | 95.55 | 1565.69 | 98.57 | 94.7 | 1008.56 | 100 | 89.42 | 233.42 | 100 | 0 |
| CBTS-16 | 587.68 | 1.27 | 376.86 | 1.16 | 368 | 10 | 91.76 | 84.05 | 22.4 | 1474.02 | 95.92 | 78.67 | 725.18 | 100 | 81.48 | 1044.8 | 100 | 89.98 | 1121.31 | 100 | 97.98 | 4333.37 | 100 | 93.44 | 1093.22 | 98.52 | 93.74 | 308 | 100 | 19.74 | 174.82 | 100 | 0 |
| CBTS-17 | 520.09 | 1.06 | 282.92 | 0.91 | 358 | 10.3 | 85.16 | 90.33 | 11.95 | 661.03 | 95.9 | 42.21 | 627.94 | 99.94 | 39.06 | 175.67 | 100 | 0 | 169.24 | 100 | 0 | 1215.5 | 100 | 63.89 | 1812.71 | 98.52 | 95.63 | 1075.16 | 100 | 74.95 | 344.55 | 100 | 16.13 |
| CBTS-18 | 781.6 | 1.78 | 468.76 | 1.55 | 336 | 9.8 | 87.01 | 78.55 | 4.1 | 291.25 | 95.92 | 15.91 | 248.79 | 100 | 0 | 316.89 | 100 | 7.01 | 131.23 | 100 | 0 | 585.81 | 100 | 42.05 | 496.62 | 98.5 | 49.42 | 282.07 | 100 | 7.88 | 126.25 | 100 | 0 |
| CBTS-19 | 574.37 | 1.25 | 311.09 | 1.07 | 317 | 10.8 | 85.91 | 90.13 | 26.78 | 1528.89 | 95.59 | 63.78 | 606.97 | 100 | 63.6 | 4563.79 | 97.82 | 90.82 | 330.74 | 99.91 | 2.77 | 4873.03 | 100 | 95.93 | 1267.83 | 98.31 | 93.97 | 984.39 | 100 | 72.29 | 12.82 | 98.14 | 0 |
| CBTS-20 | 802.41 | 1.78 | 479.34 | 1.57 | 329 | 10 | 88.22 | 84.9 | 16.15 | 1285.39 | 95.36 | 46.82 | 398.37 | 99.91 | 13.16 | 4334.17 | 95.24 | 90.58 | 303.56 | 100 | 0 | 4376.41 | 100 | 95.55 | 782.91 | 98.37 | 92.67 | 701.63 | 100 | 54.29 | 19.09 | 99.19 | 0 |
| CBTS-21 | 521.36 | 1.11 | 310.19 | 0.98 | 355 | 10.4 | 88.35 | 88.17 | 31.16 | 1757.78 | 95.65 | 67.53 | 628.94 | 99.99 | 71.96 | 5159.97 | 97.3 | 90.99 | 140.89 | 100 | 0 | 6381.94 | 100 | 98.09 | 1123.42 | 98.54 | 94.39 | 1292.91 | 100 | 91.87 | 30.24 | 100 | 0 |
| CBTS-22 | 1084.43 | 2.47 | 499.75 | 1.97 | 277 | 10.5 | 79.49 | 90.24 | 23.07 | 2317.08 | 95.94 | 67.73 | 1512.88 | 100 | 86.77 | 208.59 | 100 | 0 | 1014.73 | 100 | 97.51 | 1321.32 | 100 | 53.53 | 11981.8 | 98.67 | 97.9 | 932.17 | 100 | 64.74 | 275.94 | 100 | 10.79 |
| CBTS-23 | 914.5 | 1.65 | 597.59 | 1.5 | 479 | 11 | 90.84 | 94.03 | 33.98 | 4300.76 | 95.98 | 83.38 | 2608.85 | 100 | 98.88 | 847.8 | 100 | 82.54 | 530.42 | 100 | 72.11 | 4447.65 | 100 | 63.57 | 6562.29 | 98.91 | 97.89 | 2006.47 | 100 | 98.92 | 52950.15 | 100 | 100 |
| CBTS-24 | 892.83 | 1.74 | 546.94 | 1.57 | 392 | 10.8 | 89.89 | 95.39 | 33.21 | 3884.28 | 96.09 | 86.42 | 3012.9 | 100 | 97.82 | 4695.05 | 100 | 91.61 | 1519.18 | 100 | 99.06 | 3689.04 | 100 | 61.67 | 7513.94 | 99.64 | 97.82 | 994.06 | 100 | 83.41 | 23169.09 | 100 | 99.94 |
| CBTS-25 | 971.42 | 1.39 | 681.95 | 1.25 | 886 | 11.3 | 90.21 | 92.89 | 18.51 | 2733.4 | 95.98 | 90.21 | 2602.41 | 100 | 98.73 | 1778.11 | 100 | 99.33 | 2746.74 | 100 | 99.98 | 2210.46 | 100 | 71 | 6949.5 | 98.91 | 97.97 | 1637.31 | 100 | 99.5 | 2230.22 | 100 | 100 |
| CBTS-26 | 808.47 | 1.6 | 543.93 | 1.47 | 442 | 10.3 | 91.74 | 91.78 | 21.07 | 2390.95 | 95.94 | 86.02 | 2605.42 | 100 | 96.88 | 1178.96 | 100 | 90.39 | 797.57 | 100 | 96.62 | 3188.68 | 100 | 60.67 | 7170.36 | 98.67 | 97.85 | 1114.11 | 100 | 94.11 | 1144.58 | 100 | 97.21 |
| CBTS-27 | 735.34 | 0.63 | 344.63 | 0.43 | 1,103.00 | 8.6 | 68.14 | 90.8 | 56.54 | 2478.11 | 95.89 | 71.89 | 1683.74 | 99.87 | 92.85 | 358.72 | 98.45 | 30.13 | 406.91 | 100 | 25.58 | 3408.73 | 100 | 90.69 | 4088.14 | 99.61 | 95.37 | 572.13 | 100 | 56.12 | 21865.96 | 100 | 100 |
| CBTS-28 | 1084.18 | 1.87 | 494.23 | 1.45 | 390 | 9.5 | 77.7 | 91.59 | 18.31 | 1786.76 | 95.92 | 67.1 | 601.75 | 100 | 59.48 | 2502.38 | 100 | 90.36 | 448.97 | 100 | 26.26 | 5292.25 | 100 | 93.83 | 3194.69 | 98.49 | 96.46 | 3464.3 | 100 | 96.39 | 190.26 | 100 | 0 |
| CBTS-29 | 485.71 | 0.86 | 292.31 | 0.75 | 480 | 9.8 | 87.13 | 88.9 | 37.67 | 2083.86 | 96.08 | 82.59 | 1468.75 | 100 | 96.78 | 575.96 | 100 | 71.31 | 4706.75 | 100 | 63.76 | 3032.82 | 100 | 97.91 | 2729.09 | 99.61 | 96 | 504.54 | 100 | 37.37 | 1292.33 | 100 | 96.22 |
| CBTS-30 | 640 | 0.91 | 424.56 | 0.77 | 814 | 9.6 | 84.98 | 84.85 | 27.36 | 2070.84 | 96.1 | 87.36 | 1637.22 | 100 | 97.32 | 583.25 | 100 | 70.66 | 487.21 | 100 | 68.8 | 3880.73 | 100 | 98.6 | 3349.98 | 99.72 | 97.2 | 4091.28 | 100 | 99.96 | 5746.2 | 100 | 97.89 |
| CBTS-31 | 737.4 | 1.64 | 385.9 | 1.35 | 308 | 10.1 | 82.24 | 83.71 | 40.95 | 2553.01 | 95.1 | 68.67 | 1056.57 | 100 | 77.45 | 11150.3 | 92.71 | 91.56 | 308.02 | 99.86 | 3.84 | 6385.46 | 100 | 95.73 | 1738.97 | 98.31 | 93.3 | 1344.28 | 100 | 82.75 | 22.32 | 99.01 | 0 |
| CBTS-32 | 917.61 | 2.03 | 538.42 | 1.76 | 334 | 9.8 | 86.47 | 84.5 | 11.35 | 1042.31 | 95.92 | 61.18 | 634.44 | 100 | 56.93 | 644.67 | 100 | 85.13 | 3172.61 | 100 | 63.66 | 1086.29 | 100 | 65.07 | 1109.86 | 98.49 | 93.73 | 330.95 | 100 | 12.32 | 344.3 | 100 | 15.63 |
| CBTS-33 | 546.06 | 1.23 | 312.65 | 1.07 | 316 | 10.4 | 86.64 | 83.67 | 28.3 | 1438.29 | 95.45 | 54.12 | 467.66 | 100 | 33.89 | 4288.32 | 95.31 | 90.84 | 349.88 | 100 | 7.73 | 5224.7 | 100 | 97.43 | 910.34 | 98.49 | 92.9 | 650.6 | 100 | 46.83 | 18.97 | 100 | 0 |
| CBTS-34 | 733.18 | 1.11 | 511.82 | 1 | 917 | 10.4 | 89.69 | 84.79 | 11.02 | 1116.69 | 95.92 | 87.75 | 973.51 | 100 | 96.76 | 786.59 | 100 | 79.89 | 878.07 | 100 | 94.84 | 953.91 | 100 | 82.82 | 1826.15 | 98.54 | 96.68 | 2142.86 | 100 | 100 | 611.34 | 100 | 70.35 |
| CBTS-35 | 1151.22 | 2 | 839.08 | 1.86 | 649 | 10.8 | 93.07 | 89.59 | 15.25 | 2653.79 | 96.12 | 93.77 | 1736.82 | 100 | 98.75 | 1187.53 | 100 | 93.07 | 990.74 | 100 | 95.66 | 5537.21 | 100 | 99.93 | 3546.9 | 99.87 | 97.25 | 2585.98 | 100 | 99.46 | 1096.45 | 100 | 93.42 |
| CBTS-36 | 575.75 | 1.07 | 350.05 | 0.95 | 427 | 9.7 | 89.14 | 92.69 | 47.8 | 3223.58 | 95.86 | 82.67 | 1300.99 | 99.91 | 93.77 | 2606.89 | 100 | 100 | 5924.1 | 100 | 63.73 | 9137.84 | 100 | 98.98 | 2333 | 98.33 | 95.58 | 101.77 | 100 | 0 | 4278.22 | 100 | 100 |
| CBTS-37 | 624.43 | 1.04 | 363.8 | 0.89 | 499 | 10 | 85.63 | 92.94 | 25.71 | 1912.82 | 95.89 | 72.16 | 878.8 | 99.86 | 91.63 | 399.63 | 100 | 23.79 | 435.43 | 100 | 47.47 | 1132.26 | 100 | 71.77 | 2114.44 | 98.6 | 96.36 | 659.8 | 100 | 81.92 | 28429.2 | 100 | 100 |
| CBTS-38 | 701.01 | 1.52 | 479.69 | 1.38 | 391 | 8.7 | 90.42 | 67.41 | 7.49 | 480.37 | 95.92 | 31.69 | 365.87 | 100 | 13.04 | 375.43 | 100 | 27.91 | 425.25 | 100 | 23.04 | 479.56 | 100 | 22.17 | 941.51 | 98.5 | 93.11 | 1503.97 | 100 | 71.46 | 240.68 | 100 | 0 |
| CBTS-39 | 606.1 | 1.31 | 359.37 | 1.17 | 350 | 10.9 | 89.45 | 94 | 59.69 | 4500.03 | 96.02 | 78.02 | 2019.76 | 100 | 93.62 | 901.02 | 100 | 68.77 | 5419.9 | 100 | 99.74 | 1834.21 | 100 | 56.75 | 7856.44 | 99.2 | 97.72 | 319.3 | 100 | 14.14 | 49851.89 | 100 | 98.7 |
| CBTS-1 | 133.05 | 0.28 | 78.71 | 0.25 | 351 | 9.7 | 90.56 | 92.38 | 34.74 | 546.43 | 95.78 | 26.53 | 353.2 | 99.81 | 4.99 | 82.17 | 99.47 | 0 | 114.21 | 100 | 0 | 590.73 | 100 | 43.95 | 1064.28 | 98.33 | 91.4 | 148.63 | 100 | 0 | 5266.7 | 100 | 96.9 |
| CBTS-4 | 143.54 | 0.38 | 77.31 | 0.34 | 247 | 9.6 | 88.69 | 85.4 | 30.84 | 446.06 | 92.84 | 27.59 | 319.39 | 99.86 | 2.45 | 1279.68 | 92.61 | 52.58 | 3.29 | 83.33 | 0 | 804.43 | 100 | 51.9 | 790.5 | 98.49 | 83.89 | 102.07 | 100 | 0 | 96.39 | 100 | 0 |
| CBTS-16 | 119.67 | 0.25 | 64.26 | 0.21 | 341 | 9.6 | 85.34 | 89.43 | 22.96 | 290.68 | 95.89 | 12.7 | 144.63 | 99.94 | 0 | 202.06 | 100 | 0 | 206.75 | 100 | 0 | 855.77 | 100 | 71.48 | 213.75 | 98.44 | 0 | 59.33 | 100 | 0 | 45.94 | 100 | 0 |
| CBTS-4 | 4141 | 4.9 | 3490.75 | 4.66 | 822 | 10.2 | 95.23 | 97.76 | 0.02 | 12.87 | 79.6 | 0 | 8.7 | 92.42 | 0 | 64.18 | 91.54 | 0 | 0.34 | 25.85 | 0 | 8.59 | 97.2 | 0 | 37.94 | 98.02 | 0 | 6.37 | 64.5 | 0 | 2.64 | 90.32 | 0 |
| CBTS-22 | 6353.21 | 7.62 | 4714.78 | 6.86 | 759 | 11.2 | 89.94 | 97.13 | 0.03 | 25.43 | 79.97 | 0 | 27.64 | 95.57 | 0 | 0.38 | 28.96 | 0 | 13.06 | 99.02 | 0 | 9.21 | 79.84 | 0 | 150.45 | 98.86 | 0 | 12.75 | 83.62 | 0 | 0.76 | 25.31 | 0 |

**Supplementary Table 4. Fusion detected in the standard samples.**

| Validation phase | Sample ID | Expected fusion | Detected fusion | 5' gene breakpoint | 3' gene breakpoint | Number of support_reads | 5' gene breakpoint depth | 3' gene breakpoint depth | Fusion rate(%) |
| --- | --- | --- | --- | --- | --- | --- | --- | --- | --- |
| BCR-ABL1-standard-NGS | BCR-ABL1-NGS1 | BCR::ABL1 | BCR::ABL1 | chr22:23289621 | chr9:130854064 | 7580 | 9842.05 | 26151.7 | 28.98% |
| BCR-ABL1-standard-NGS | BCR-ABL1-NGS2 | BCR::ABL1 | BCR::ABL1 | chr22:23289621 | chr9:130854064 | 8035 | 10768.7 | 27225 | 29.51% |
| BCR-ABL1-standard-short reads | BCR-ABL1-QNome-short | BCR::ABL1 | BCR::ABL1 | chr22:23289618 | chr9:130854061 | 1230 | 3169.05 | 7807.95 | 15.75% |
| BCR-ABL1-standard-short reads | BCR-ABL1-QNome-short | BCR::ABL1 | BCR::ABL1 | chr22:23289619 | chr9:130854061 | 2294 | 4510.2 | 13586.25 | 16.88% |
| BCR-ABL1-clinical-short reads | CCPVS-1 | ETV6::PDGFRB | ETV6::PDGFRB | chr12:11853559 | chr5:150126612 | 50 | 653.85 | 1964.4 | 2.55% |
| BCR-ABL1-clinical-short reads | CCPVS-2 | BCR::ABL1 | BCR::ABL1 | chr22:23182235 | chr9:130854061 | 7 | 48.35 | 1207 | 0.58% |
| BCR-ABL1-standard-long reads | BCR-ABL1-QNome-long | BCR::ABL1 | BCR::ABL1 | chr22:23289619 | chr9:130854063 | 2214 | 7828.4 | 10358.15 | 21.37% |
| BCR-ABL1-clinical-long reads | CCPVS-1 | ETV6::PDGFRB | ETV6::PDGFRB | chr12:11853559 | chr5:150126613 | 1891 | 3507.7 | 11541.4 | 16.38% |
| BCR-ABL1-clinical-long reads | CCPVS-2 | BCR::ABL1 | BCR::ABL1 | chr22:23182236 | chr9:130854061 | 140 | 428.5 | 2998.55 | 4.67% |
| Input test | CCPVS-2 | BCR::ABL1 | BCR::ABL1 | chr22:23182236 | chr9:130854061 | 140 | 428.5 | 2998.55 | 4.67% |
| Input test | CCPVS-2 | BCR::ABL1 | BCR::ABL1 | chr22:23182237 | chr9:130854060 | 112 | 299.45 | 2260.8 | 4.95% |
| Input test | CCPVS-2 | BCR::ABL1 | BCR::ABL1 | chr22:23182236 | chr9:130854062 | 119 | 322.7 | 3027.65 | 3.93% |
| Input test | CCPVS-2 | BCR::ABL1 | BCR::ABL1 | chr22:23182237 | chr9:130854061 | 160 | 330.05 | 2637.65 | 6.07% |
| Input test | CCPVS-2 | BCR::ABL1 | BCR::ABL1 | chr22:23182237 | chr9:130854060 | 446 | 805.75 | 5391.8 | 8.27% |
| Input validation | CCPVS-2 | BCR::ABL1 | BCR::ABL1 | chr22:23182237 | chr9:130854060 | 568 | 836.55 | 5341.7 | 10.63% |
| Input validation | CCPVS-2 | BCR::ABL1 | BCR::ABL1 | chr22:23182237 | chr9:130854061 | 117 | 242.4 | 1860.05 | 6.29% |
| Input validation | CCPVS-2 | BCR::ABL1 | BCR::ABL1 | chr22:23182236 | chr9:130854060 | 90 | 249.45 | 2601.45 | 3.46% |
| Input validation | CCPVS-2 | BCR::ABL1 | BCR::ABL1 | chr22:23182237 | chr9:130854060 | 65 | 160.35 | 1224.1 | 5.31% |
| Input validation | CCPVS-2 | BCR::ABL1 | BCR::ABL1 | chr22:23182236 | chr9:130854061 | 140 | 236.9 | 1834.6 | 7.63% |
| Input validation | CCPVS-2 | BCR::ABL1 | BCR::ABL1 | chr22:23182236 | chr9:130854060 | 80 | 204.5 | 1544.65 | 5.18% |
| PCR condition | CCPVS-3 | BCR::ABL1 | - | - | - | - | - | - | - |
| PCR condition | CCPVS-4 | BCR::ABL1 | BCR::ABL1 | chr22:23290412 | chr9:130854069 | 5 | 18.35 | 979.1 | 0.51% |
| PCR condition | CCPVS-5 | BCR::ABL1 | BCR::ABL1 | chr22:23289617 | chr9:130854061 | 12 | 43.05 | 638.6 | 1.88% |
| PCR condition | CCPVS-3 | BCR::ABL1 | - | - | - | - | - | - | - |
| PCR condition | CCPVS-6 | BCR::ABL1 | - | - | - | - | - | - | - |
| PCR condition | CCPVS-4 | BCR::ABL1 | BCR::ABL1 | chr22:23290411 | chr9:130854065 | 14 | 60.9 | 2771.1 | 0.51% |
| PCR condition | CCPVS-7 | BCR::ABL1 | BCR::ABL1 | chr22:23289620 | chr9:130854061 | 16 | 26.55 | 1871.65 | 0.85% |
| PCR condition | CCPVS-5 | BCR::ABL1 | BCR::ABL1 | chr22:23289619 | chr9:130854063 | 32 | 85.7 | 1484.95 | 2.15% |
| PCR condition | CCPVS-8 | BCR::ABL1 | BCR::ABL1 | chr22:23290410 | chr9:130854064 | 185 | 378.5 | 1463.1 | 12.64% |
| PCR condition | CCPVS-3 | BCR::ABL1 | - | - | - | - | - | - | - |
| PCR condition | CCPVS-6 | BCR::ABL1 | - | - | - | - | - | - | - |
| PCR condition | CCPVS-4 | BCR::ABL1 | - | - | - | - | - | - | - |
| PCR condition | CCPVS-7 | BCR::ABL1 | - | - | - | - | - | - | - |
| PCR condition | CCPVS-5 | BCR::ABL1 | BCR::ABL1 | chr22:23289619 | chr9:130854062 | 37 | 90.35 | 1466.05 | 2.52% |
| PCR condition | CCPVS-8 | BCR::ABL1 | BCR::ABL1 | chr22:23290411 | chr9:130854064 | 180 | 532.85 | 1970.4 | 9.14% |

| SampleID | Expected fusion | LongGF | fusionseeker | jaffal | genion |
| --- | --- | --- | --- | --- | --- |
| BCR-ABL1-QNome-short1 | BCR::ABL1 | BCR::ABL1 | BCR::ABL1 | BCR::ABL1 | - |
| BCR-ABL1-QNome-short1 | BCR::ABL1 | BCR::ABL1 | BCR::ABL1 | BCR::ABL1 | - |
| BCR-ABL1-QNome-long | BCR::ABL1 | BCR::ABL1 | - | BCR-ABL1 | - |
| CCPVS-2 | BCR::ABL1 | BCR::ABL1 | BCR::ABL1 | - | - |
| CCPVS-1 | ETV6::PDGFRB | ETV6::PDGFRB | ETV6::PDGFRB | ETV6::PDGFRB | - |
| CCPVS-2 | BCR::ABL1 | BCR::ABL1 | - | BCR::ABL1 | - |
| CCPVS-1 | ETV6::PDGFRB | ETV6::PDGFRB | - | ETV6::PDGFRB | - |

**Supplementary Table 5. Fusion detection performance evaluation across 4 tools**

*Light blue background indicates unexpected fusion detected in that sample.

**Supplementary Table 6. Fusion detection using PACLseq, and current clinical detection method turnaround time and cost for blinded samples.**

| **GroupA** | Expected fusion | Detected fusion | 5' gene breakpoint | 3' gene breakpoint | Number of support_reads | 5' gene breakpoint depth | 3' gene breakpoint depth | Fusion rate(%) | RIN | Turnaround  Time of current detection method (d) | RT-PCR expense (CNY) (Fusion Gene Panel for Leukimia) | FISH expense (CNY) | RNA-seq expense (CNY) | RT-PCR expense (CNY) (Fusion Gene Panel for Ph-like ALL) | RT-PCR expense (CNY)  (Fusion Gene Panel for ALL) | Total Expense of current detection method(CNY) |
| --- | --- | --- | --- | --- | --- | --- | --- | --- | --- | --- | --- | --- | --- | --- | --- | --- |
| CBTS-1 | P2RY8-CRLF2 | P2RY8-CRLF2 | chrX:1536919 | chrX:1212637 | 11654 | 20002.55 | 23906.1 | 48.75% | 3.3 | 8 | 2000 | 4500 | 0 | 1500 | 0 | 8000 |
| CBTS-2 | P2RY8-CRLF2 | P2RY8-CRLF2 | chrX:1536919 | chrX:1212636 | 46 | 59.95 | 485.65 | 9.47% | 4.4 | 16 | 2000 | 4500 | 5000 | 0 | 0 | 11500 |
| CBTS-3 | P2RY8-CRLF2 MYO18B-ABL1 | P2RY8-CRLF2 | chrX:1536919 | chrX:1212636 | 2890 | 5506.55 | 7921.25 | 36.48% | 4 | 19 | 2000 | 4500 | 5000 | 0 | 0 | 11500 |
| CBTS-3 | P2RY8-CRLF2 MYO18B-ABL1 | MYO18B-ABL1 | chr22:25955360 | chr9:130862762 | 309 | 599.85 | 2645.65 | 11.68% | 4 | 19 | 2000 | 4500 | 5000 | 0 | 0 | 11500 |
| CBTS-4 | SSBP2-CSF1R | SSBP2-CSF1R | chr5:81615473 | chr5:150061846 | 249 | 1453.2 | 3159.05 | 7.88% | 8 | 9 | 2000 | 0 | 0 | 1500 | 0 | 3500 |
| CBTS-5 | negative | - | - | - | - | - | - | - | 7.6 | - | - | - | - | - | - | - |
| CBTS-6 | P2RY8/CRLF2 | P2RY8-CRLF2 | chrX:1536919 | chrX:1212635 | 134 | 234.7 | 669.1 | 20.03% | 3.9 | 12 | 2000 | 4500 | 0 | 1500 | 0 | 8000 |
| CBTS-7 | NUP214-ABL1 | NUP214-ABL1 | chr9:131199013 | chr9:130854060 | 24 | 70.45 | 1368.65 | 1.75% | 3.1 | 12 | 0 | 4500 | 5000 | 0 | 0 | 9500 |
| CBTS-8 | P2RY8-CRLF2 | P2RY8-CRLF2 | chrX:1536919 | chrX:1212636 | 3292 | 5460.75 | 8734.1 | 37.69% | 8 | 16 | 2000 | 4500 | 5000 | 0 | 0 | 11500 |
| CBTS-9 | negative | - | - | - | - | - | - | - | 6.8 | - | - | - | - | - | - | - |
| CBTS-10 | negative | - | - | - | - | - | - | - | 4.7 | - | - | - | - | - | - | - |
| CBTS-11 | negative | - | - | - | - | - | - | - | 5.9 | - | - | - | - | - | - | - |
| CBTS-12 | negative | - | - | - | - | - | - | - | 6.4 | - | - | - | - | - | - | - |
| CBTS-13 | negative | - | - | - | - | - | - | - | 7.3 | - | - | - | - | - | - | - |
| CBTS-14 | negative | - | - | - | - | - | - | - | 7.3 | - | - | - | - | - | - | - |
| CBTS-15 | ETV6/ABL1 | - | - | - | - | - | - | - | 4.5 | 8 | 0 | 0 | 0 | 1500 | 0 | 1500 |
| CBTS-16 | PAX5-JAK2 | PAX5-JAK2 | chr9:37002647 | chr9:5081722 | 2345 | 4119.45 | 8783.05 | 26.70% | 3.8 | 17 | 2000 | 0 | 5000 | 0 | 0 | 7000 |
| CBTS-17 | P2RY8-CRLF2 | P2RY8-CRLF2 | chrX:1536919 | chrX:1212636 | 70 | 84.95 | 281.1 | 24.90% | 5.1 | 20 | 0 | 4500 | 5000 | 0 | 0 | 9500 |
| CBTS-18 | P2RY8/CRLF2 | P2RY8-CRLF2 | chrX:1536919 | chrX:1212636 | 40 | 44 | 118.2 | 33.84% | 3.2 | 13 | 2000 | 4500 | 0 | 1500 | 0 | 8000 |
| CBTS-19 | negative | - | - | - | - | - | - | - | 8.1 | - | - | - | - | - | - | - |
| CBTS-20 | negative | - | - | - | - | - | - | - | 5.1 | - | - | - | - | - | - | - |
| CBTS-21 | negative | - | - | - | - | - | - | - | 8 | - | - | - | - | - | - | - |
| CBTS-22 | NUP214-ABL1 | NUP214-ABL1 | chr9:131222927 | chr9:130854061 | 164 | 241 | 9931.55 | 1.65% | 4.2 | 12 | 2000 | 0 | 5000 | 0 | 0 | 7000 |
| CBTS-23 | P2RY8-CRLF2 | P2RY8-CRLF2 | chrX:1536919 | chrX:1212636 | 21805 | 33493.7 | 53163.1 | 41.02% | 4.1 | 15 | 2000 | 4500 | 5000 | 0 | 0 | 11500 |
| CBTS-24 | P2RY8-CRLF2 | P2RY8-CRLF2 | chrX:1536919 | chrX:1212636 | 9826 | 14535.85 | 20672.8 | 47.53% | 4.4 | 22 | 2000 | 4500 | 5000 | 0 | 0 | 11500 |
| CBTS-25 | ZBTB5-JAK2 | ZBTB5-JAK2 | chr9:37441386 | chr9:5064881 | 624 | 858 | 2651.85 | 23.53% | 4.3 | 15 | 0 | 4500 | 5000 | 1500 | 1200 | 12200 |
| CBTS-26 | negative |  |  |  |  |  |  |  | 4.1 | - | - | - | - | - | - | - |
| **GroupB** | | | | | | | | | | | | | | | | |
| CBTS-27 | P2RY8-CRLF2 | P2RY8-CRLF2 | chrX:1536920 | chrX:1212636 | 3728 | 8977.5 | 15824 | 23.56% | 2.50 | 8 | 2000 | 4500 | 0 | 1500 | 0 | 8000 |
| CBTS-28 | NUP214-ABL1 | - | - | - | - | - | - | - | 3 | 16 | 0 | 0 | 5000 | 0 | 0 | 5000 |
| CBTS-29 | EBF1/PDGFRB | EBF1-PDGFRB | chr5:158712155 | chr5:150126612 | 1281 | 2157.8 | 4388.75 | 29.19% | not detected | 13 | 0 | 0 | 0 | 1500 | 0 | 1500 |
| CBTS-30 | P2RY8-CRLF2 | P2RY8-CRLF2 | chrX:1536919 | chrX:1212635 | 1864 | 2873.55 | 5075.85 | 36.72% | not detected | 15 | 2000 | 0 | 5000 | 0 | 0 | 7000 |
| CBTS-31 | negative | - | - | - | - | - | - | - | 3 | - | - | - | - | - | - | - |
| CBTS-34 | P2RY8-CRLF2 | - | - | - | - | - | - | - | not detected | 23 | 2000 | 0 | 5000 | 1500 | 0 | 8500 |
| CBTS-32 | EBF1-PDGFRB | EBF1-PDGFRB | chr5:158712155 | chr5:150126612 | 784 | 1687.3 | 2938.35 | 26.68% | not detected | 14 | 2000 | 4500 | 5000 | 0 | 0 | 11500 |
| CBTS-33 | negative | - | - | - | - | - | - | - | 2.6 | - | - | - | - | - | - | - |
| CBTS-35 | PAX5/JAK2 | PAX5-JAK2 | chr9:37002647 | chr9:5081723 | 2723 | 4504.55 | 8508.25 | 3200.40% |  | 15 | 0 | 4500 | 0 | 1500 | 0 | 6000 |
| **GroupC** | | | | | | | | | | | | | | | | |
| CBTS-36 | Possible rearrangement of PDGFRB and CSF1R | EBF1-PDGFRB | chr5:158707978 | chr5:150126614 | 2662 | 4618.35 | 7346.3 | 36.24% | 7.6 | 16 | 0 | 4500 | 0 | 1500 | 1200 | 7200 |
| CBTS-37 | Possible rearrangement of CRLF2 | CRLF2 overexpression | - | - | - | - | - | - | 3.5 | 6 | 2000 | 4500 | 0 | 0 | 0 | 6500 |
| CBTS-38 | IGH/EPOR | ENSG00000277856:EPOR | KI270726.1:26533 | chr9:11378232 | 276 | - | 707.38 | 39.02% | 3.6 | 14 | 2000 | 0 | 0 | 1500 | 0 | 3500 |
| CBTS-39 | Possible rearrangement of CRLF2 | CRLF2 overexpression | - | - | - | - | - | - | 5.4 | 6 | 0 | 4500 | 0 | 0 | 0 | 4500 |
| **Multiplex-test** | | | | | | | | | | | | | | | | |
| CBTS-1 | P2RY8-CRLF2 | P2RY8-CRLF2 | chrX:1536919 | chrX:1212636 | 1902 | 3250.8 | 4128.4 | 46.07% | - | - | - | - | - | - | - | - |
| CBTS-4 | SSBP2-CSF1R | SSBP2-CSF1R | chr5:81615473 | chr5:150061847 | 120 | 614.4 | 1435.95 | 8.36% | - | - | - | - | - | - | - | - |
| CBTS-16 | PAX5-JAK2 | PAX5-JAK2 | chr9:37002647 | chr9:5081722 | 436 | 789.35 | 1706 | 25.56% | - | - | - | - | - | - | - | - |

**Supplementary Material and methods:**

**I PACLseq Protocol**

PACLseq is a method designed to capture and sequence long-fragment cDNA. The process involves several steps, including:

1. RNA quantification and size distribution analysis;

2. RNA fragmentation;

3. cDNA synthesis;

4. pre-capture illumina library preparation;

5. target capture with a customized panel;

6. post-capture nanopore library preparation;

7. QNome nanopore sequence;

8. Bioinfomatic analysis;

**II FISH Protocol**

**III mRNA-Seq Protocol**

**IV RNA Extraction and Quantification**

**I PACLseq Protocol**

**Mainly used reagent**:

Nuclease-free Water( AM9930, Thermo Fisher Scientific)

VAHTS® DNA Clean Beads(N411-03, Vazyme)

Equalbit 1×dsDNA HS Assay Kit(EQ121-02, Vazyme)

mRNA-seq Lib Prep Kit for illumina(RK20302, ABclonal)

2X Frag/Elute Buffer

RT Reagent

First Strand Synthesis Enzyme Mix

Second Strand Synthesis Reaction Buffer

Second Strand Synthesis Enzyme Mix

Nuclease-free Water

End-prep Buffer

End-prep Enzyme Mix

Ligation Buffer

Ligase Mix

2X PCR Mix

Low EDTA TE

RNA Adapter Module 96 Index for Illumina(RK20351/RK20352, ABclonal)

TargetSeq® Target Probes(iGeneTech, PT1008252)

TargetSeq® Target Probes

TargetSeq® Cap Beads & Nuclease-Free Water(iGeneTech, C10422)

TargetSeq® Cap Beads

Nuclease-Free Water

TargetSeq One® Hyb & Wash Kit with Eco Universal Blocking Oligo(iGeneTech, C10732)

TargetSeq One® Hyb & Wash Kit (Module A)

Hyb Human Block

RNase Block

TargetSeq One® Hyb Buffer

TargetSeq One® Hyb & Wash Kit (Module B)

Binding Buffer

TargetSeq One® Wash Buffer

Wash Buffer 1

TargetSeq One® Hyb & Wash Kit (Module C, for Illumina)

Post PCR Master Mix

Post PCR Primer (25 μM)*

TargetSeq® Eco Universal Blocking Oligo (for Illumina)

**QLK-V1.1.1(Qitan Tech)**

Sequencing adapter(SAC)

4x ligation buffer(LRB)

long fragment wash buffer(LWB)

short fragment wash buffer(SWB)

control DNA sequence(CDS)

elution buffer(AEB)

DNA ligaition enzyme(DLE)

DNA repair enzyme(DRM)

DNA repair buffer(DRB)

end repair enzyme(EPM)

**QSK-V1.1.1(Qitan Tech )**

**1.RNA quantification and size distribution analysis**

RNA quantification and size distribution analysis are conducted using the Agilent Tapestation 4150 system. Subsequently, 1ul mRNA is quantified using the qubit method.

**2.mRNA fragmentation**

The mRNA is chemically fragmented in 2X Frag/Elute Buffer at elevated temperatures. The fragmentation process is carried out under the following conditions:

| mRNA input | x* ng |
| --- | --- |
| 2X Frag/Elute Buffer | 5ul |
| Total volume(add Nuclease-free Water) | 10ul |
| Processing | 94℃, 5min |

*****In this work, we tested the procedure using concentrations of 10 ng, 20 ng, 30 ng, 50 ng, and 100 ng.

**3.cDNA synthesis**

After the mRNA fragmentation, the next steps involve first strand cDNA synthesis and second strand cDNA synthesis. These steps collectively enable the conversion of fragmented mRNA into double-stranded cDNA, facilitating further downstream applications such as library construction and sequencing.

First strand cDNA synthesis:

| mRNA(previous step) | 10ul |
| --- | --- |
| RT Reagent volume | 8ul |
| First Stand Synthesis Enzyme Mix | 2ul |
| Total volume | 20ul |
| Processing | 25℃, 10min;  42℃, 30min;  70℃, 15min; |

Subsequently, second strand cDNA synthesis is carried out, and the synthesized product is purified using VAHTS DNA Clean Beads to remove any unwanted impurities or residual reaction components.

| first strand cDNA product(previous step) | 20ul |
| --- | --- |
| Second Strand Synthesis Reaction Buffer | 8ul |
| Second Strand Synthesis Enzyme Mix | 4ul |
| Total volume(add Nuclease-free Water) | 80ul |
| Processing | 16℃, 2h |
| Purification(VAHTS DNA Clean Beads) | 1.8x |

**4.Pre-capture illumina library preparation**

After mRNA fragmentation, the resulting product undergoes library construction (Figure S1).

First, the cDNA fragments undergo end-repair, where any damaged or uneven ends are repaired to generate blunt ends. This is important for ensuring optimal ligation efficiency in the subsequent steps.

| Purified cDNA product(previous step) | 37ul |
| --- | --- |
| End-prep Buffer | 10ul |
| End-prep Enzyme Mix | 3ul |
| Total volume | 50ul |
| Processing | 30℃,30min;  65℃,30min; |

Next, the repaired cDNA fragments are ligated with an RNA Truncated adapter. The adapter contains sequences necessary for downstream processes.

| End-repaired an purified cDNA product(previous step) | 50ul |
| --- | --- |
| Ligation Buffer | 16.5ul |
| RNA Truncated Adapter | 2.5ul |
| Ligase Mix | 3ul |
| Processing | 22℃,30min; |
| Purification(VAHTS DNA Clean Beads) | 0.45x |

Following the ligation with the RNA Truncated adapter, a Universal PCR adapter is ligated to the cDNA fragments and purified. This adapter allows for the amplification of the cDNA fragments in the subsequent PCR step.The ligated product, containing both the RNA Truncated adapter and the Universal PCR adapter, is subjected to PCR amplification using primers specific to the universal adapter sequences. After PCR amplification, the amplified product is purified and ready for the subsequent target capture step.

| RNA Truncated adaptered product (previous step) | 20ul |
| --- | --- |
| 2×PCR Mix | 25ul |
| Universal PCR Primer | 2.5ul |
| PCR Index Primer | 2.5ul |
| PCR amplification processing | 98℃45s;  98℃ 10s;  60℃ 15s;  72℃ 1min; |
| PCR cycle(different cycle for different input) | 10ng-1:11  10ng-2:20  20ng:18  30ng:18  50ng:16  100ng:13 |
| Processing | 72℃ 5min; |
| Purification(VAHTS DNA Clean Beads) | 1x |

**5.Target capture with a customized panel**

Target capture with a customized panel is conducted by setting up a reaction system with the appropriate conditions. The previous product is quantified, and 750ng of the product is concentrated for subsequent use. The mixture is gently mixed and incubated for 3 minutes. Subsequently, 28ul of the supernatant is combined with 2ul of the Target Probe and incubated for 16 hours.

| TargetSeq One Hyb Buffer | 13ul |
| --- | --- |
| Hyb Human Block | 5ul |
| TargetSeq Eco Universal Blocking Oligo | 2ul |
| Rnase Block | 5ul |
| Total volume(add Nuclease-free Water) | 28ul |

**6.Post-capture nanopore library preparation**

In the post-capture nanopore library preparation step, cap beads are prepared and added to the hybridized product to selectively absorb the hybridized DNA fragments. After thorough washing, the cap beads with the absorbed DNA fragment are subjected to post-capture PCR under specific conditions.

| Component | For 10ng mRNA input | Other mRNA input |
| --- | --- | --- |
| previous products | 25ul | 25ul |
| Post PCR Master Mix | 2.5ul | 2.5ul |
| Post PCR Primer | 2.5ul | 2.5ul |
| PCR amplification processing | 95℃ 3min;  98℃ 20s;  60℃ 30s;  72℃ 1min; | 95℃ 1min;  98℃ 20s;  60℃ 30s;  72℃ 30s; |
| PCR cycle(different cycle for different input) | 10ng-1:18  10ng-2:20 | 16 |
| Processing | 72℃ 5min; | 72℃ 5min; |
| Purification(VAHTS DNA Clean Beads) | 1.1x | 1.1x |

After the PCR amplification step, the product is purified and end-repaird, then 300fmol product is used to construct the nanopore library following QLK-V1.1.1 protocol(QitanTech).

| cDNA | 300fmol |
| --- | --- |
| DRB | 7ul |
| EPM | 3ul |
| Total volume(add Nuclease-free Water) | 60ul |
| processing | 20℃10min;  65℃10min; |
| Purification(VAHTS DNA Clean Beads) | 1x |
| End-repaired products | 60ul |
| SAC | 5ul |
| LRB | 25ul |
| DLE | 10ul |
| hold still 10min | |
| Purification(VAHTS DNA Clean Beads) | 0.4x |
| AEB | 15ul |

**7.QNome nanopore sequence**

The product (300fmol) underwent end-repair and nanopore adapter ligation using the QNome-3841 (122mm*126mm*48mm, 0.8kg, Figure S2) library preparation protocol (QLK-V1.1.1, QitanTech). The 80fmol nanopore adapter ligated product is loaded into a nanopore cell for sequencing about 16h following the QSK-V1.1.1 protocol.

1. **Bioinfomatic analysis**

For illumina short read data, fastqc was uased to do quality control and star-fusion was used to detect fusions, the running example

singularity exec -e -B /path \

        /softpath/star-fusion.v1.12.0.simg \

        STAR-Fusion \

        --left_fq /datapath/left.fq \

        --right_fq /datapath/right.fq \

        --genome_lib_dir /softpath/source/ctat_genome_lib_build_dir \

        --no_remove_dups \

        -O /outpath/sample

For nanopore data, data quality control, reference alignment and LongGF fusion detection and filtering were described in our streamlined fusion detection pipeline([https://github.com/HuanYuu/TargetFusion](https://github.com/HuanYuu/TargetFusion).)). Running examples for other fusion detection method were described as follow.

Fusionseeker running example:

fusionseeker \

--bam /datapath/sample.sorted.bam \

--datatype nanopore \

--maxdistance 40 \

--minsupp 5 \

--thread 10 \

--keepfile \

--outpath /outpath/sample_fusionseeker \

--ref /referencepath/GRCh38.fa \

--gtf /referencepath/Homo_sapiens.GRCh38.104.chrname.gtf.gz

JAFFAL running example:

/softpath/tools/bin/bpipe run \

-n 10 \

/softpath/JAFFAL.groovy \

/fastqpath/sample.fq.gz

Genion running example:

genion \

-i sample.fq.gz \

--gtf /softpath/Genion_dependent/Homo_sapiens.GRCh38.97.gtf \

--gpaf /datapath/sample.sorted.paf \

-s /softpath/Genion_dependent/hg38_cdna.selfalign.tsv \

-d /softpath/Genion_dependent/genomicSuperDups.txt \

-o /outpath/sample.fusion.tsv \

--non-coding

Among the four softwares, LongGF got the highest recall rate (77.78%) and the result was more suitble for customized filtering. The log files generated by LongGF, containing detailed read span information, were utilized for custom filtering to further improve precision. The following filters were applied to the LongGF outputs and the results were presented in Table S4:

1. The fusion gene pairs must be joined together in the 5'-3' direction.

2. The average depth of the targeted gene's breakpoint (20bp) must be greater than or equal to 100.

3. The fusion rate, calculated by dividing the number of fusion-supported reads by the average depth of the target captured gene breakpoint (20bp), must be larger than 0.5%.

4. The number of supported fusion reads must be at least 5.

5. The average 20bp breakpoint depth of the partner gene must be higher than the number of fusion-supported reads.

**II FISH Protocol**

FISH was performed using probes targeting PDGFRB, CSF1R, CRLF2, ABL1, ABL2, JAK2, and EPOR. A total of 400 cells were analyzed per probe. The probes were supplied by Wuhan HealthCare Biotechnology Co., Ltd.

Reference：

1) PMID: 26314984：Fluorescence in situ hybridization (FISH) analysis was performed on interphase nuclei at diagnosis from directly harvested peripheral blood or bone marrow samples according to the manufacturer's protocol and using the following commercially available probes (Abbott Molecular, Des Plaines, IL, USA): LSI MYB (6q23), LSI P53 (17p13.1)/ LSI ATM (11q22.3), LSI D13S319 (13q14.3)/CEP12, as reported. A minimum of 400 nuclei were scored for each probe or probe combination.

2) PMID: 35912172：FISH analyses were performed according to our institutional protocols ( 10 ). Accordingly, a commercial panel of FISH probes covering Philadelphia chromosome-like B-lymphoblastic leukemia (Ph-like ALL), including ABL1 , ABL2 , CRLF2 , EPOR , and JAK2 , was purchased (Vysis, Abbott/IL/USA). A positive rearrangement was reported when at least 3% of the nuclei showed break-apart split signals. Bone marrow (BM) samples at diagnosis or relapse were analyzed by FISH (Olympus IX 71, Tokyo, Japan).

**III mRNA-Seq Protocol**

RNA Sequencing Platform：Illumina MiSeq system (Illumina MiSeq, USA)

Sequencing reads were aligned to the UCSC hg19 reference genome using STAR software. Variant calling was performed with VarDict, and gene fusion events were predicted using STAR-Fusion.

Reference：

1) PMID: 27869790：Transcriptome libraries were sequenced on Illumina HiSeq 2000 or MiSeq machines (Supplementary Table 19).

2) PMID: 33481105：Cluster generation and sequencing were then performed on the MiSeq System (Illumina).

3) PMID: 32393662：Sequenced reads were aligned to the UCSC hg19 reference genome assembly and gene counts were quantified using STAR (v2.5.1b) 19.

4) PMID: 33743195：Variant calling and annotation were respectively performed using the VarDict and ANNOVAR software tools.

5) PMID: 29789628：For gene fusion detection, we use STAR-Fusion (https://github.com/STAR-Fusion/STAR-Fusion).

**IV RNA Extraction and Quantification:**

Total RNA was extracted using the RNAiso Plus reagent (TaKaRa, Dalian, China). RNA concentration and purity were assessed using a NanoDrop spectrophotometer (Thermo Fisher Scientific, Waltham, MA, USA).

Reference：

1) PMID: 30518811：Total RNA were extracted by RNAiso Plus reagent (TaKaRa, Dalian, China).

2) PMID: 33523829：RNA isolation was performed using the RNAiso Plus (TAKARA) according to the manufacturer’s instructions.

3) PMID: 35594351：RNA was quantified with a NanoDrop spectrophotometer (Thermo Fisher Scientific, Waltham, MA, USA).

4) PMID: 35420131：DNA concentration was quantified using a NanoDrop spectrophotometer (Thermo Fisher Scientific, Inc., Waltham, MA, USA).

References

1. Roberts KG, Li Y, Payne-Turner D, et al. Targetable kinase-activating lesions in Ph-like acute lymphoblastic leukemia. 2014;371(11):1005-1015.

2. Roberts KG, Gu Z, Payne-Turner D, et al. High Frequency and Poor Outcome of Philadelphia Chromosome-Like Acute Lymphoblastic Leukemia in Adults. *Journal of clinical oncology : official journal of the American Society of Clinical Oncology.* 2017;35(4):394-401.

3. Boer JM, Steeghs EM, Marchante JR, et al. Tyrosine kinase fusion genes in pediatric BCR-ABL1-like acute lymphoblastic leukemia. *Oncotarget.* 2017;8(3):4618-4628.

4. Imamura T, Kiyokawa N, Kato M, et al. Characterization of pediatric Philadelphia-negative B-cell precursor acute lymphoblastic leukemia with kinase fusions in Japan. *Blood cancer journal.* 2016;6(5):e419.

5. Reshmi SC, Harvey RC, Roberts KG, et al. Targetable kinase gene fusions in high-risk B-ALL: a study from the Children's Oncology Group. *Blood.* 2017;129(25):3352-3361.

6. Chang F, Lin F, Cao K, et al. Development and Clinical Validation of a Large Fusion Gene Panel for Pediatric Cancers. *J Mol Diagn.* 2019;21(5):873-883.

7. Poukka M, Lund-Aho T, Raittinen P, et al. Acute Lymphoblastic Leukemia With INPP5D-ABL1 Fusion Responds to Imatinib Treatment. *Journal of pediatric hematology/oncology.* 2019;41(7):e481-e483.

8. Kakadia PM, Tizazu B, Mellert G, et al. A novel ABL1 fusion to the SH2 containing inositol phosphatase-1 (SHIP1) in acute lymphoblastic leukemia (ALL). *Leukemia.* 2011;25(10):1645-1649.

9. He GQ, Lei YP, Huang DW, Gao J, Yang R. Philadelphia chromosome-like acute lymphoblastic leukemia with concomitant rearrangements of CRLF2 and ABL1: a pediatric case report. *BMC Pediatr.* 2024;24(1):517.

10. Tan KW, Zhu YY, Qiu QC, et al. Rapid molecular response to dasatinib in Ph-like acute lymphoblastic leukemia patients with ABL1 rearrangements: case series and literature review. *Ann Hematol.* 2023;102(9):2397-2402.

11. Oya S, Morishige S, Ozawa H, et al. Beneficial tyrosine kinase inhibitor therapy in a patient with relapsed BCR-ABL1-like acute lymphoblastic leukemia with CCDC88C-PDGFRB fusion. *International journal of hematology.* 2021;113(2):285-289.

12. Severson EA, Vergilio JA, Gay LM, et al. Genomic Landscape of Adult and Pediatric BCR-ABL1-Like B-Lymphoblastic Leukemia Using Parallel DNA and RNA Sequencing. *The oncologist.* 2019;24(3):372-374.

13. Xu G, Bao X-b, Liu L-m, et al. Genetic Profile and Clinical Implications of PDGFRB Fusion in Adult B-Cell Acute Lymphoblastic Leukemia: A Retrospective Analysis. 2021.

14. Panagopoulos I, Brunetti M, Stoltenberg M, et al. Novel GTF2I-PDGFRB and IKZF1-TYW1 fusions in pediatric leukemia with normal karyotype. *Experimental hematology & oncology.* 2019;812.

15. Zhang X, Hou Z, Huang D, et al. Single-cell heterogeneity and dynamic evolution of Ph-like acute lymphoblastic leukemia patient with novel TPR-PDGFRB fusion gene. *Experimental hematology & oncology.* 2023;12(1):19.

16. Sadras T, Jalud FB, Kosasih HJ, et al. Unusual PDGFRB fusion reveals novel mechanism of kinase activation in Ph-like B-ALL. *Leukemia.* 2023;37(4):905-909.

17. Liu YF, Wang BY, Zhang WN, et al. Genomic Profiling of Adult and Pediatric B-cell Acute Lymphoblastic Leukemia. *EBioMedicine.* 2016;8173-183.

18. Yano M, Imamura T, Asai D, et al. Identification of novel kinase fusion transcripts in paediatric B cell precursor acute lymphoblastic leukaemia with IKZF1 deletion. *British journal of haematology.* 2015;171(5):813-817.

19. Mullighan CG, Collins-Underwood JR, Phillips LA, et al. Rearrangement of CRLF2 in B-progenitor–and Down syndrome–associated acute lymphoblastic leukemia. 2009;41(11):1243-1246.

20. Yano M, Imamura T, Asai D, et al. An overall characterization of pediatric acute lymphoblastic leukemia with CRLF2 overexpression. *"Genes, chromosomes & cancer".* 2014;53(10):815-823.

21. Zhang XY, Dai HP, Li Z, et al. Identification of STRBP as a Novel JAK2 Fusion Partner Gene in a Young Adult With Philadelphia Chromosome-Like B-Lymphoblastic Leukemia. *Frontiers in oncology.* 2020;10611467.

22. Lee WY, Pfau RB, Choi SM, et al. The diagnostic challenges and clinical course of a myeloid/lymphoid neoplasm with eosinophilia and ZBTB20-JAK2 gene fusion presenting as B-lymphoblastic leukemia. *Cold Spring Harbor molecular case studies.* 2020;6(2).

23. Roberts KG, Payne-Turner D, McCastlain K, et al. High Frequency and Poor Outcome of Ph-like Acute Lymphoblastic Leukemia in Adults. *Blood.* 2015;126(23):2618-2618.

24. Kawamura M, Taki T, Kaku H, Ohki K, Hayashi Y. Identification of SPAG9 as a novel JAK2 fusion partner gene in pediatric acute lymphoblastic leukemia with t(9;17)(p24;q21). *"Genes, chromosomes & cancer".* 2015;54(7):401-408.

25. Chen X, Wang F, Zhang Y, et al. Identification of RNPC3 as a novel JAK2 fusion partner gene in B-acute lymphoblastic leukemia refractory to combination therapy including ruxolitinib. *Molecular genetics & genomic medicine.* 2020;8(3):e1110.

26. Downes CEJ, Rehn J, Heatley SL, Yeung D, McClure BJ, White DL. Identification of a novel GOLGA4-JAK2 fusion gene in B-cell acute lymphoblastic leukaemia. *British journal of haematology.* 2022;196(3):700-705.

27. Tran TH, Langlois S, Meloche C, et al. Whole-transcriptome analysis in acute lymphoblastic leukemia: a report from the DFCI ALL Consortium Protocol 16-001. *Blood advances.* 2022;6(4):1329-1341.

28. Iacobucci I, Li Y, Roberts KG, et al. Truncating Erythropoietin Receptor Rearrangements in Acute Lymphoblastic Leukemia. *Cancer cell.* 2016;29(2):186-200.

29. Gu Z, Churchman M, Roberts K, et al. Genomic analyses identify recurrent MEF2D fusions in acute lymphoblastic leukaemia. *Nature communications.* 2016;713331.

30. Barnes EJ, Leonard J, Medeiros BC, Druker BJ, Tognon CE. Functional characterization of two rare BCR-FGFR1(+) leukemias. *Cold Spring Harbor molecular case studies.* 2020;6(2).

31. Zhang Z, Zhu Y, Wang Z, et al. Case Report: A novel FGFR1 fusion in acute B-lymphoblastic leukemia identified by RNA sequencing. *Frontiers in oncology.* 2023;131276695.

32. Trimaldi J, Carballido EM, Bowers JW, et al. B-lymphoblastic leukemia/lymphoma associated with t(8;13)(p11;q12)/ ZMYM2 (ZNF198)-FGFR1 : rare case and review of the literature. *Acta haematologica.* 2013;130(3):127-134.
